# Multiple large language models versus experienced physicians in diagnosing challenging cases with gastrointestinal symptoms

**DOI:** 10.1038/s41746-025-01486-5

**Published:** 2025-02-05

**Authors:** Xintian Yang, Tongxin Li, Han Wang, Rongchun Zhang, Zhi Ni, Na Liu, Huihong Zhai, Jianghai Zhao, Fandong Meng, Zhongyin Zhou, Shanhong Tang, Limei Wang, Xiangping Wang, Hui Luo, Gui Ren, Linhui Zhang, Xiaoyu Kang, Jun Wang, Ning Bo, Xiaoning Yang, Weijie Xue, Xiaoyin Zhang, Ning Chen, Rui Guo, Baiwen Li, Yajun Li, Yaling Liu, Tiantian Zhang, Shuhui Liang, Yong Lv, Yongzhan Nie, Daiming Fan, Lina Zhao, Yanglin Pan

**Affiliations:** 1 State Key Laboratory of Holistic Integrative Management of Gastrointestinal Cancers and National Clinical Research Center for Digestive Diseases, Xijing Hospital of Digestive Diseases, Fourth Military Medical University, Xi’an, China; 2 Department of Pathology, Union Hospital, Tongji Medical College, Huazhong University of Science and Technology, Wuhan, China; 3 Department of Gastroenterology, Xiamen Humanity Hospital, Fujian Medical University, Xiamen, China; 4 Department of Gastroenterology, Hainan General Hospital (Hainan Affiliated Hospital of Hainan Medical University), Haikou, China; 5 Department of Gastroenterology, Xuanwu Hospital, Capital Medical University, Beijing, China; 6 Department of Gastroenterology, Huaihe Hospital of Henan University, Kaifeng, China; 7 Department of Gastroenterology, Beijing Friendship Hospital, Capital Medical University, National Clinical Research Center for Digestive Disease, Beijing Digestive Disease Center, Beijing Key Laboratory for Precancerous Lesion of Digestive Disease, Beijing, China; 8 Department of Gastroenterology, Renmin Hospital of Wuhan University, Wuhan, China; 9 Department of Gastroenterology, The General Hospital of Western Theater Command, Chengdu, China; 10 Department of Gastroenterology, Shaanxi Second Provincial People’s Hospital, Xi’an, China; 11 Department of Gastroenterology, The 986th Hospital of Xijing Hospital, Air Force Military Medical University, Xi’an, China; 12 Department of Gastroenterology, The Second Affiliated Hospital of Chongqing Medical University, Chongqing, China; 13 Department of Gastroenterology, Zhongshan Hospital of Xiamen University, School of Medicine, Xiamen University, Xiamen, China; 14 Department of Transplantation and Pediatric Surgery, Kumamoto University Hospital, Kumamoto, Japan; 15 Department of Gastroenterology, National Clinical Research Center of Infectious Disease, The Third People’s Hospital of Shenzhen, The Second Affiliated Hospital of Southern University of Science and Technology, Shenzhen, China; 16 Department of Gastroenterology, Peking University People’s Hospital, Peking University, Beijing, China; 17 Department of Gastroenterology, Beijing Shijingshan Hospital, Capital Medical University, Beijing, China; 18 Department of Gastroenterology, Shanghai General Hospital, Shanghai Jiao Tong University School of Medicine, Shanghai, China; 19 Department of Gastroenterology, General Hospital of Ningxia Medical University, Yinchuan, China; 20 Department of Radiotherapy, Xijing Hospital, Fourth Military Medical University, Xi’an, China

**Keywords:** Diagnosis, Computational models

## Abstract

Faced with challenging cases, doctors are increasingly seeking diagnostic advice from large language models (LLMs). This study aims to compare the ability of LLMs and human physicians to diagnose challenging cases. An offline dataset of 67 challenging cases with primary gastrointestinal symptoms was used to solicit possible diagnoses from seven LLMs and 22 gastroenterologists. The diagnoses by Claude 3.5 Sonnet covered the highest proportion (95% confidence interval [CI]) of instructive diagnoses (76.1%, [70.6%–80.9%]), significantly surpassing all the gastroenterologists (*p* < 0.05 for all). Claude 3.5 Sonnet achieved a significantly higher coverage rate (95% CI) than that of the gastroenterologists using search engines or other traditional resource (76.1% [70.6%–80.9%] vs. 45.5% [40.7%-50.4%], *p* < 0.001). The study highlights that advanced LLMs may assist gastroenterologists with instructive, time-saving, and cost-effective diagnostic scopes in challenging cases.

## Introduction

Undiagnosed diseases are a widespread issue. It is estimated that undiagnosed diseases affect approximately 30 million Americans and are associated with high rates of morbidity and mortality^1^. These patients often undergo prolonged and costly diagnostic odysseys involving repeated and redundant diagnostic efforts, which carry risks of invasive procedures and false diagnoses^2^. Complex disorders with atypical or nonspecific symptoms can pose diagnostic challenges for physicians^1,3,4^. Confronted with challenging cases, physicians may develop diagnostic biases due to limitations in their routine clinical experience. To reach a diagnosis, physicians frequently need to consult additional literature, which can be inefficient and time-consuming. Consequently, challenging cases often require multidisciplinary discussions^5,6^. However, high-level multidisciplinary teams (MDTs) are limited and often costly^7^. Therefore, effective and accessible diagnostic support is urgently needed for the diagnosis of challenging cases.

With the rapid development of large language models (LLMs), closed-source LLMs such as GPT-4, Gemini-1.5-pro, and Claude 3.5 Sonnet have become widely adopted among individual users. Physicians are also increasingly using these tools in clinical work^8–10^. In the clinical field, researchers have found that LLMs can pass the United States Medical Licensing Examination^11^, highlighting their potential in medical knowledge. Compared to human specialists, LLMs have demonstrated a broad understanding of multiple medical domains, suggesting their potential as versatile diagnostic tools.

In diagnosing challenging cases, some researchers have investigated the capabilities of LLMs in undiagnosed or complex cases^12–14^. Eriksen et al. conducted a survey involving 38 online challenging cases among medical journal readers, revealing that GPT-4’s diagnostic performance surpassed that of most of the readers^12^. However, online cases increase the risk of information leakage and lead to potential bias^15^. Some other researchers used fictional cases to explore GPT-4’s diagnostic value^16^. In contrast, Shea et al. evaluated GPT-4’s diagnostic abilities using six closed-source real-world challenging cases, suggesting that GPT-4 may alert clinicians to important missed diagnoses^13^. However, the limited sample size (*n* = 6) constrains the generalizability of the findings. Ethan et al. reported the diagnostic accuracy of 25 physicians using GPT-4 did not significantly surpass that of another 25 physicians using traditional auxiliary methods^17^. The study was well-designed but still had several limitations. The study only evaluated one type of LLM, included a small number of cases (*n* = 6), and relied on relatively inexperienced physicians (median years in practice = 3). Real-world challenging cases generally do not seek help from less experienced physicians, which limits the reference value of the study.

Current research on LLMs in medical diagnosis faces several significant limitations. First, some researchers used fictional cases, which cannot fully capture the complexity of actual patient presentations^16^. Although real-world case studies exist, they are often limited by small sample sizes and information leakage, while also lacking comprehensive comparisons between LLMs and experienced specialist physicians^12,16,17^. Furthermore, comprehensive evaluations of different LLMs’ diagnostic capabilities remain scarce, despite the crucial role of accurate differential diagnoses in challenging cases. The comparative effectiveness of different LLMs vs. traditional diagnostic aids, such as search engines and literature databases, also remains largely unexplored. Perhaps most critically, there has been insufficient analysis of potential risks associated with LLM use in diagnosis, including diagnostic errors and hallucinations. Given the increasing use of LLMs in consulting on challenging cases, addressing these research gaps has become increasingly urgent.

To explore the above issues and address the limitations of existing studies, it was necessary to establish an offline dataset of challenging real cases. Over the past five years, cases involving gastrointestinal (GI) symptoms accounted for approximately 20% of the Case Records of the Massachusetts General Hospital, published in the New England Journal of Medicine (NEJM) (see Supplementary Table 1). Given the diversity of clinical departments, this proportion is relatively large. Apart from GI diseases, many disorders, such as hyperthyroidism and multiple myeloma, can involve atypical GI symptoms, leading patients to seek care from GI departments^18,19^. These cases can pose diagnostic challenges for gastroenterologists. Therefore, challenging cases with GI symptoms provide an ideal touchstone for testing LLMs and gastroenterologists. Moreover, considering that our researchers specialize in GI, we chose to establish an offline dataset of challenging cases whose primary symptoms included nonspecific GI symptoms.

In this study, we established an offline dataset to ask seven widely used LLMs and 22 experienced gastroenterologists to offer possible diagnoses and the most likely diagnosis. The gastroenterologists were allowed to use traditional diagnostic aids (such as search engines and literature databases). The possible diagnoses and the most likely diagnoses given by LLMs or physicians were evaluated. To further analyze the feasibility of applying LLMs in clinical workflow, we compared the diagnostic time and costs between LLMs and physicians. Additionally, we conducted detailed analyses of hallucinations and diagnostic errors. Through these tests and analyses, this study aims to explore the potential role of LLMs in supporting the diagnosis of challenging real-world cases.

## Results

### Dataset

**Fig. 1:**
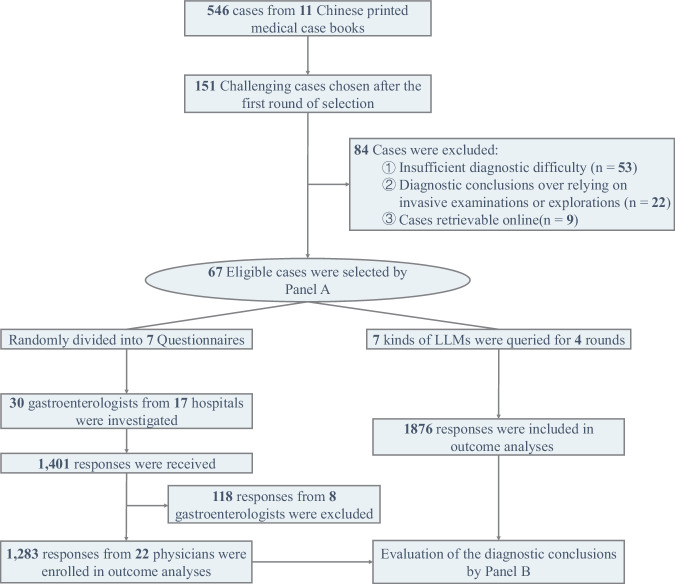
Flow diagram of the study. This flowchart outlines the selection and evaluation process. From 546 cases in 11 Chinese medical case books, 151 challenging cases were chosen. After excluding 84 cases due to insufficient difficulty (*n* = 53), reliance on invasive diagnostics (*n* = 22), or online retrievability (*n* = 9), 67 eligible cases were selected by Panel A and divided into seven questionnaires. Surveys from 22 gastroenterologists (17 hospitals) yielded 1283 valid responses. Seven LLMs provided 1876 responses over four rounds, with diagnostic conclusions analyzed and compared.

The flowchart is shown in Fig. 1. From a total of 546 cases found in 11 medical case books (Supplementary Table 2), 151 challenging cases were initially selected after the first round of evaluation. Subsequently, 84 cases were excluded due to insufficient diagnostic difficulty, over-reliance on invasive examinations for diagnostic conclusions, or cases that could be retrieved online. Ultimately, 67 challenging cases were included in the dataset, comprising 25 gastrointestinal (GI) cases and 42 non-GI cases (Supplementary Table 3). The average token number (standard deviation [SD]) of the case records was 575.3 (175.4). The dataset was randomly divided into 7 questionnaires for distribution.

### Diagnostic outcomes

Seven widely used closed-source LLMs, including GPT-3.5t, GPT-4o, Gemini-1.0-pro, Gemini-1.5-pro, Claude-2.1, Claude 3 Opus and Claude 3.5 Sonnet, were evaluated through four rounds of repeat queries. Each query was conducted independently, without any prior conversation history. In contrast, thirty experienced gastroenterologists participated in this survey. The median (IQR) clinical experience in GI of the 22 physicians was 18.5 (12.0–22.8) years.

**Fig. 2:**
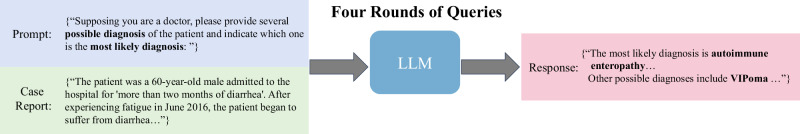
Query pipeline of the LLMs. The content of the question consists of two parts a prompt to ask LLMs to provide several possible diagnoses and one most likely diagnosis, and a detailed case report. Each case was individually queried four times.

As instructed, the seven LLMs answered all questions and generated 1876 responses (Figs. 1 and 2). Thirty gastroenterologists were recruited and submitted 1401 responses (Fig. 1). Eight physicians with low response rates (13.4%–29.9%) were excluded because they answered less than 40% of the questions (27/67). The remaining 22 physicians had an 87.0% (1283/1474) response rate, and the 1283 responses were subsequently included in the analysis. Of the 1283 submitted responses, 2.7% were left blank. The LLMs generated an average of 4.4–5.6 possible diagnoses per question (Table 1), significantly higher than the physicians, who generated an average of 1.4 possible diagnoses (*p* < 0.001).

**Table 1:** Diagnostic performance of LLMs in four rounds

Model	Average number of diagnoses (SD^a^)	Coverage rate (%, 95% CI^a^)	Accuracy (%, 95% CI^a^)	Krippendorff’s Alpha
**GPT-3.5t**	4.4(1.0)	22.8 (18.1–28.2)	6.0 (3.7–9.5)	0.643
Round 1	4.6(1.1)	25.4 (15.9–37.5)	4.5 (1.2–12.7)	
Round 2	4.4(1.1)	19.4 (11.2–31.0)	7.5 (2.9–16.7)	
Round 3	4.4(0.9)	17.9 (10.0–29.3)	3.0 (0.6–10.5)	
Round 4	4.2(1.0)	28.4 (18.4–40.7)	9.0 (3.8–18.6)	
**GPT-4o**	5.3(1.0)	64.2 (58.2–69.8)	42.9 (37.0–49.0)	0.664
Round 1	5.4(1.0)	56.7 (44.1–68.6)	35.8 (24.8–48.4)	
Round 2	5.4(1.0)	62.7 (50.1–73.9)	43.3 (31.4–55.9)	
Round 3	5.3(0.9)	68.7 (56.2–79.1)	47.8 (35.6–60.2)	
Round 4	5.2(0.9)	68.7 (56.2–79.1)	44.8 (32.8–57.3)	
**Gemini-1.0-pro**	5.3(1.3)	30.6 (25.3–36.4)	16.4 (12.4–21.4)	0.555
Round 1	5.3(1.5)	31.3 (20.9–43.8)	17.9 (10.0–29.3)	
Round 2	5.4(1.3)	32.8 (22.2–45.4)	19.4 (11.2–31.0)	
Round 3	5.3(1.3)	26.9 (17.1–39.1)	11.9 (5.7–22.3)	
Round 4	5.2(1.2)	31.3 (20.9–43.8)	16.4 (8.9–27.6)	
**Gemini-1.5-pro**	5.2(0.9)	53.4 (47.3–59.3)	24.6 (19.8–30.2)	0.720
Round 1	5.2(0.8)	53.7 (41.3–65.8)	22.4 (13.5–34.3)	
Round 2	5.2(0.8)	53.7 (41.3–65.8)	28.4 (18.4–40.7)	
Round 3	5.3(0.8)	52.2 (39.8–64.4)	23.9 (14.7–35.9)	
Round 4	5.1(0.9)	53.7 (41.3–65.8)	23.9 (14.7–35.9)	
**Claude-2.1**	4.6(0.8)	40.3 (34.5–46.4)	22.4 (17.7–27.8)	0.656
Round 1	4.6(0.9)	38.8 (27.4–51.4)	20.9 (12.3–32.6)	
Round 2	4.6(0.8)	41.8 (30.1–54.4)	23.9 (14.7–35.9)	
Round 3	4.5(0.8)	43.3 (31.4–55.9)	22.4 (13.5–34.3)	
Round 4	4.5(0.7)	37.3 (26.1–49.9)	22.4 (13.5–34.3)	
**Claude 3 Opus**	4.8(0.6)	66.4 (60.5–71.9)	44.4 (38.5–50.5)	0.697
Round 1	4.8(0.7)	64.2 (51.6–75.2)	47.8 (35.6–60.2)	
Round 2	4.8(0.6)	68.7 (56.2–79.1)	41.8 (30.1–54.4)	
Round 3	4.9(0.6)	65.7 (53.1–76.5)	44.8 (32.8–57.3)	
Round 4	4.8(0.5)	67.2 (54.6–77.8)	43.3 (31.4–55.9)	
**Claude 3.5 Sonnet**	5.6(0.7)	76.1 (70.6–80.9)	48.9 (42.9–54.9)	0.780
Round 1	5.5(0.6)	77.6 (65.7–86.5)	47.8 (35.6–60.2)	
Round 2	5.6(0.7)	77.6 (65.7–86.5)	49.3 (37.0–61.6)	
Round 3	5.7(0.7)	77.6 (65.7–86.5)	52.2 (39.8–64.4)	
Round 4	5.7(0.7)	71.6 (59.3–81.6)	46.3 (34.2–58.7)	

^a^*SD* standard deviation, *CI* confidence interval.

**Fig. 3:**
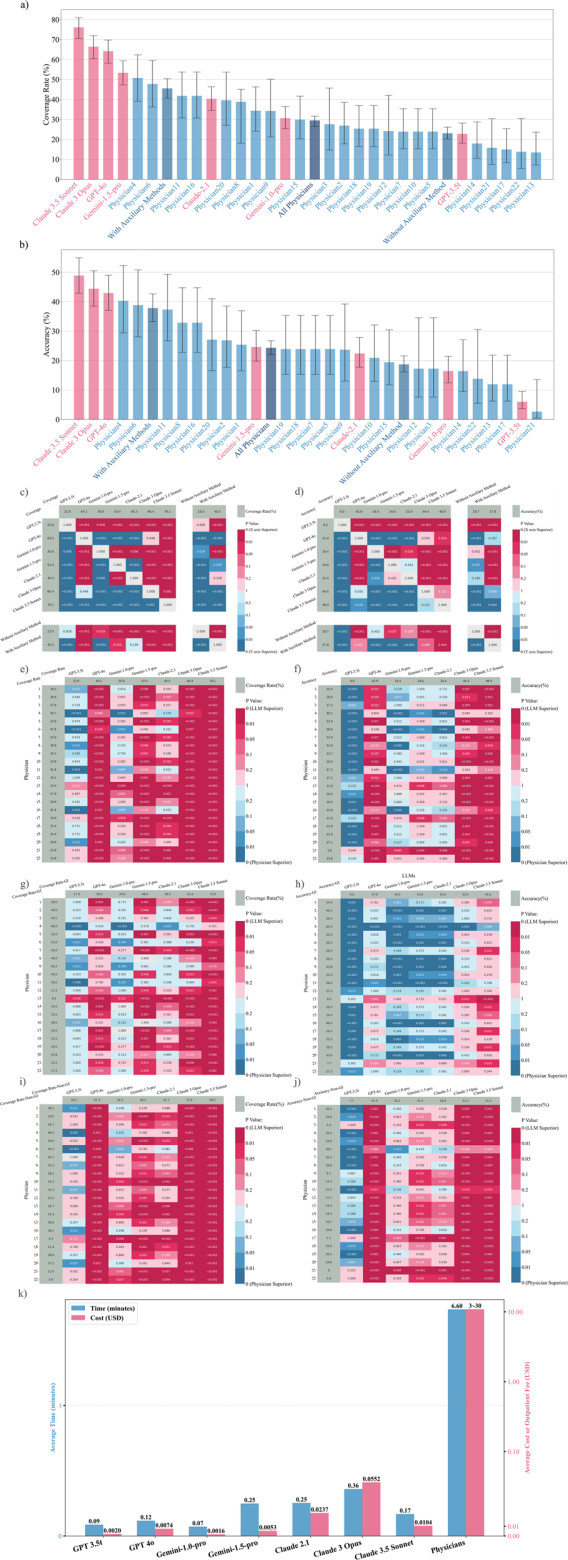
Diagnostic performance and time-cost analysis of the LLMs and the physicians. **a** The bar chart illustrates the ranking of coverage rates for different LLMs and physicians. **b** The bar chart illustrates the ranking of accuracies for different LLMs and physicians. **c**, **d** The left heatmap shows the coverage rates and the right heatmap shows the accuracies of each LLM compared to physicians using or not using auxiliary methods. Corresponding statistical significance is shown in each heatmap. **e**, **f** The left heatmap displays the coverage rates, and the right heatmap presents the accuracies of each LLM compared to physicians, along with their corresponding statistical significance. **g**, **h** The left heatmap shows the coverage rates and the right heatmap displays the accuracies of each LLM vs. physicians in the GI case subgroup, including the corresponding statistical significance. Claude 3.5 Sonnet had the highest coverage rates. Apart from Claude 3.5 Sonnet, Physician 4 significantly outperformed other LLMs in diagnostic accuracy for GI cases. **i**, **j** The left heatmap illustrates the coverage rates and the right heatmap shows the accuracies of each LLM compared to physicians in the non-GI case subgroup, along with their corresponding statistical significance. **k** The bar chart compares the average time taken to answer a single question by seven different LLMs and the 22 physicians. It also presents a cost comparison between the fee charged by the LLMs for answering a single question and the cost range for a single outpatient visit to one of the 22 experienced physicians (excluding examination and treatment fees).

Coverage rate was used to evaluate the proportion of responses whose possible diagnoses included correct diagnosis or instructive diagnosis (see “Methods” section for details). Across the four rounds, the coverage rates of the LLMs, ranked from highest to lowest, were as follows: Claude 3.5 Sonnet (76.1%), Claude 3 Opus (66.4%), GPT-4o (64.2%), Gemini-1.5-pro (53.4%), Claude-2.1 (40.3%), Gemini-1.0-pro (30.6%), and GPT-3.5t (22.8%) (Fig. 3a and Table 1). Claude 3.5 Sonnet surpassed the other 6 LLMs (*p* < 0.05 for all) (Fig. 3c). The physicians’ coverage rates ranged from 13.4% to 50.7%, with an average coverage rate (SD, 95% confidence interval [CI]) of 29.5% (10.7%, 27.1%-32.1%) (Fig. 3a and Supplementary Table 4). The coverage rates of Claude 3.5 Sonnet and Claude 3 Opus were significantly higher than those of all physicians (22/22, *p* < 0.05 for all). GPT-4o’s coverage rate was significantly higher than that of 95.5% (21/22, *p* < 0.05) of the physicians (Fig. 3e). The statistical significance of differences between 7 LLMs in each round and the physicians were detailed in Supplementary Figs. 1 and 2.

Accuracy was defined as the rate at which the most likely diagnoses were consistent or mostly consistent with the actual diagnoses (see “Methods” section for details). The accuracies of the LLMs were as follows: Claude 3.5 Sonnet (48.9%), Claude 3 Opus (44.4%), GPT-4o (42.9%), Gemini-1.5-pro (24.6%), Claude-2.1 (22.4%), Gemini-1.0-pro (16.4%) and GPT-3.5t (6.0%) (Fig. 3b and Table 1). The statistical differences in accuracy between different LLMs are shown in Fig. 3d. The coverage rate of Claude 3.5 Sonnet (76.1%) was significantly higher than its accuracy (48.9%, *p* < 0.001) (Supplementary Fig. 3). The accuracy of the physicians ranged from 2.6% to 40.3%, with an average accuracy (SD) of 24.3% (9.2%, 95% CI: 22.0–26.7%). Claude 3.5 Sonnet, Claude 3 Opus, and GPT-4o demonstrated accuracies that were significantly superior to 86.3% (19/22), 77.3% (17/22), and 72.7% (16/22) of physicians, respectively (Fig. 3f).

The coverage rate of the physicians was positively correlated with the average time spent on each question (correlation coefficient = 0.474, *p* = 0.026). The physicians’ accuracy was positively correlated with their age (correlation coefficient = 0.542, *p* = 0.009) and years of clinical experience (correlation coefficient = 0.449, *p* = 0.036) (Supplementary Table 5). Using auxiliary diagnostic methods was positively correlated with coverage and accuracy, with correlation coefficients of 0.522 and 0.538, respectively (*p* = 0.013 and *p* = 0.010). The token number of case questions was not significantly correlated with the diagnostic performance of the LLMs (Supplementary Table 6).

To evaluate the consistency of LLMs, Krippendorff’s Alpha was calculated. Krippendorff’s Alpha of each LLM in four rounds of queries ranged from 0.555 to 0.780 (Table 1). Claude 3.5 Sonnet reached the highest Krippendorff’s Alpha (0.780), followed by Gemini-1.5-pro (0.720) and Claude 3 Opus (0.697).

### Subgroup analysis

Our dataset comprises 25 GI and 42 non-GI cases. The non-GI cases include immunology (*n* = 10), hematology (*n* = 9), multisystem disorders (*n* = 9), genetics (*n* = 8), and others (*n* = 6) (Supplementary Table 3).

In the GI subgroup, Claude 3.5 Sonnet achieved the highest coverage rate (73.0%), followed by Physician 4 (68.0%) and GPT-4o (59.0%) (Fig. 3g). Physician 4 achieved the highest accuracy (64.0%), followed by Physician 11 (48.0%) and Claude 3.5 Sonnet (45.0%) (Fig. 3h). Claude 3.5 Sonnet’s coverage rate exceeded that of 90.9% (20/22) of the gastroenterologists. Detailed information is shown in Supplementary Fig. 3 and Supplementary Tables 4, 7. Although the accuracy of Claude 3.5 Sonnet (45.0%) was lower than Physician 4 (64.0%, *p* = 0.089), the accuracy of Claude 3.5 Sonnet significantly outperformed the overall accuracy of the 22 physicians (31.6%, *p* = 0.014).

In the non-GI subgroup, Claude 3.5 Sonnet had the highest coverage rate (78.0%), surpassing all participating physicians (*p* < 0.05 for all) (Fig. 3i). Claude 3.5 Sonnet and Claude 3 Opus both achieved the highest accuracy (51.2%) which was significantly higher than that of 95.5% participating physicians (21/22) (Fig. 3j). The LLMs had significantly higher overall coverage rate and accuracy in non-GI cases than those in GI cases (54.2% vs. 44.3% and 34.4% vs. 20.9%; *p* < 0.001 for both). In contrast, the 22 physicians achieved a better coverage rate and accuracy in GI cases than those in non-GI cases (36.2% vs. 25.6% and 31.6% vs. 20.0%; *p* < 0.001 for both).

For physicians, we found that the 402 answers using auxiliary methods had significantly higher coverage rates and accuracy than the 786 answers not using such methods (45.5% vs. 23.0% and 37.8% vs. 18.7%, *p* < 0.001 for both) (Fig. 3c, d and Table 2). Claude 3.5 Sonnet, Claude 3 Opus, GPT-4o, and Gemini-1.5-pro significantly outperformed the physicians not using auxiliary methods in both coverage and accuracy (*p* < 0.05 for all). On the other hand, Claude 3.5 Sonnet, Claude 3 Opus, GPT-4o, and Gemini-1.5-pro had significantly higher coverage than the physicians using auxiliary methods (*p* < 0.05 for all). However, only Claude 3.5 Sonnet outperformed the physicians using auxiliary methods in accuracy (48.9% vs. 37.8%, *p* = 0.004).

**Table 2:** Diagnostic performance of the gastroenterologists using different numbers of auxiliary methods

Auxiliary methods	Number of answers	Refusal rate (%, 95% CI)	Coverage rate (%, 95% CI)	Accuracy (%, 95% CI)
Not using auxiliary methods	786	0.4 (0.1–1.1)	23.0 (20.2–26.1)	18.7 (16.1–21.6)
Using auxiliary methods	402	0.3 (0.04–1.4)	45.5 (40.7–50.4)	37.8 (33.2–42.7)
One method	299	0 (0–1.3)	48.8 (43.2–54.5)	41.1 (35.7–46.8)
1. Discussion with Peers in Gastroenterology	16	0 (0–19.4)	56.3 (33.2–76.9)	50.0 (28.0–72.0)
2. Consultation with Peers in Other Departments	4	0 (0–49.0)	100.0 (34.2–100.0)	100.0 (34.2–100.0)
3. Professional Books Reference	50	0 (0–7.1)	42.0 (29.4–55.8)	36.0 (24.1–49.9)
4. Classical Search Engine	87	0 (0–4.2)	51.7 (41.4–61.9)	42.5 (32.7–53.0)
5. Academic Database	138	0 (0–2.7)	49.3 (41.1–57.5)	41.3 (33.4–49.7)
6. Others^a^	4	0 (0–56.2)	25.0 (6.2–79.2)	25.0 (6.2–79.2)
Two methods	93	1.1 (0.2–5.8)	34.4 (25.6–44.5)	26.9 (18.9–36.7)
Three or more methods	10	0 (0–27.8)	50.0 (23.7–76.3)	40.0 (16.8–68.7)
No record of the using of auxiliary methods	95	31.6 (23.1–41.5)	15.8 (9.8–24.4)	13.7 (8.2–22.0)
Total	1283	0.3 (0.1–0.9)	29.5 (27.1–32.1)	24.3 (22.0–26.7)

^a^LLMs or search engines with LLMs function were not allowed.

### Analysis of time and cost

To evaluate the time spent per question using different numbers of auxiliary methods, we built a multiple linear regression model (Supplementary Note 1). The estimated time spent per case without any auxiliary method and with one, two, and three auxiliary methods, was 3.93, 8.74, 11.76, and 17.94 min, respectively. It should be noted that these estimates were based on the average effect of the number of methods rather than specific types, and individual auxiliary methods may vary in time requirements. Compared with the average response time of LLMs (0.19 min) (Fig. 3k, detailed in Supplementary Table 8), the overall average time spent per case using traditional auxiliary methods (6.60 min) appears to be more time-consuming. It should be noted that the average response time of LLMs was based on the response time of accessing LLMs through APIs. When users interact with LLMs via chat interfaces, additional time is required for input and reading, which may result in longer overall interaction times.

The average cost per diagnostic response varied among different LLMs (Fig. 3k). Claude 3 Opus was the most expensive at 0.0552 USD, while GPT 3.5t (0.0020 USD) and Gemini-1.0-pro (0.0016 USD) were the least expensive. Considering performance factors, Claude 3.5 Sonnet may offer the best cost-effectiveness (0.0104 USD) among the seven LLMs. The consultation fee for a specialist outpatient visit (excluding examination fees) with the 22 physicians ranged from approximately 3 USD to 30 USD. Therefore, from a cost perspective alone, the integration of advanced LLMs as diagnostic support tools may not significantly increase consultation costs.

### Hallucination analysis

The inter-rater reliability for the two rounds of hallucination analysis yielded a kappa coefficient of 0.906. After further discussion, the raters reached a consensus on 81 initially disputed cases. The final analysis of 1876 responses by the LLMs across four rounds revealed that 685 responses contained hallucinations (detailed in Supplementary Table 9). Among the 7 LLMs, Claude 3.5 Sonnet exhibited the lowest rate of hallucinations, with 57 instances (21.3%, 95% CI: 16.7–26.6%). In contrast, Gemini-1.5-pro exhibited the highest rate of hallucinations, with 168 instances (62.7%, 95% CI: 56.7%–68.3%).

**Fig. 4:**
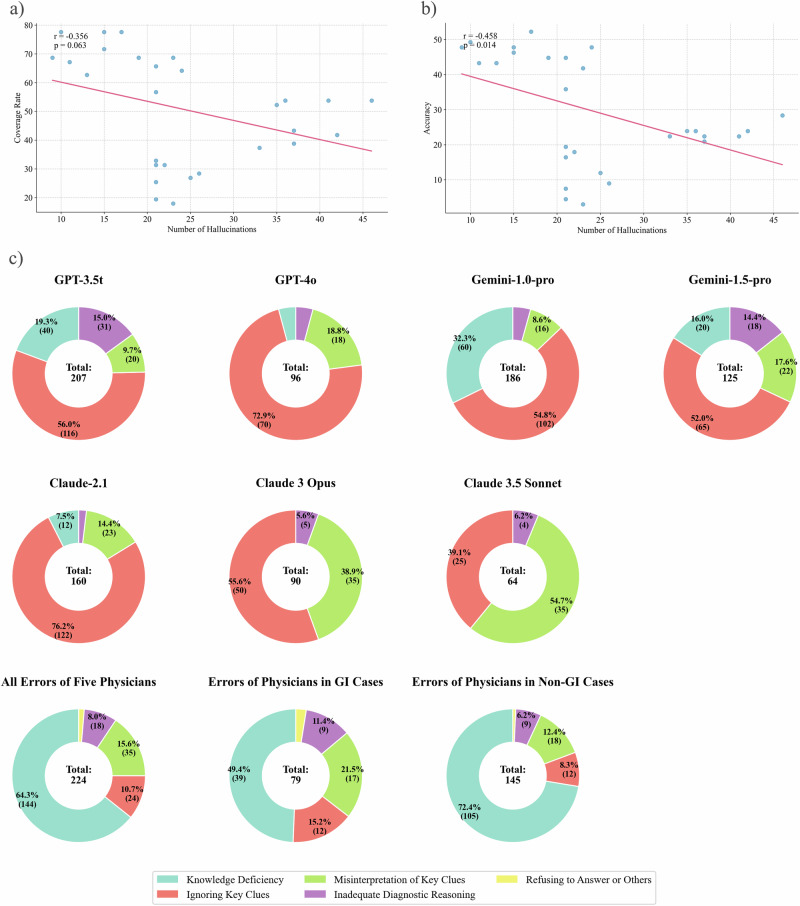
Analysis of hallucinations in the LLMs and classification of diagnostic errors by LLMs and physicians. **a**, **b** The left scatter plot shows the distribution of the number of hallucinations vs. the coverage rate for each round of responses by seven LLMs. A negative correlation was observed but not statistically significant (Pearson coefficient = −0.356, *p* = 0.063). The right scatter plot displays the distribution of the number of hallucinations vs. accuracy for each round of responses by the same LLMs, revealing a significant negative correlation (Pearson coefficient = −0.458, *p* = 0.014). **c** The first seven pie charts illustrate the categorization of error types for the seven LLMs across four rounds of repeated testing. The last three pie charts represent the error categorization made by five physicians: one for all cases in the dataset, one for the GI subgroup, and one for the non-GI subgroup.

For each round and each LLM, we analyzed the correlation between the number of hallucinations and the diagnostic performance. The results revealed a moderate negative correlation between the number of hallucinations and the accuracy (Pearson correlation coefficient r = -0.458, *p* = 0.014) (Fig. 4b). Similarly, a moderate but not statistically significant negative correlation was observed between the number of hallucinations and the coverage rate (*r* = −0.356, *p* = 0.063) (Fig. 4a).

To further investigate the relationship between the presence of hallucinations and the occurrence of response errors, we conducted a correlation analysis on instances where hallucinations co-occurred with incorrect answers in each response. The Phi coefficient for different LLMs ranged from 0 to 0.128. Among all LLMs, only GPT-3.5t showed significant results in the coverage error analysis. For this specific case, the Phi coefficient was statistically significant (0.128, *p* = 0.039), and the odds ratio analysis yielded statistically significant results (odds ratio = 0.516, *p* = 0.026). This implies that for GPT-3.5t, responses containing hallucinations might be associated with a lower probability of correct answers in terms of coverage rate. All other cases demonstrated non-significant Phi coefficients and odds ratios (Supplementary Table 10). Despite the single significant result for GPT-3.5t, these findings suggest a very weak or possibly non-existent correlation between the presence of hallucinations and response errors across most of the LLMs.

### Analysis of erroneous diagnoses

In the evaluation of responses, those classified as Category 3 were considered erroneous (see Methods for classification details). A total of 928 incorrect responses from the 7 LLMs were identified across four rounds of questioning. The reasons for erroneous responses were categorized into five groups: Knowledge Deficiency, Ignoring Key Clues, Misinterpretation of Key Clues, Inadequate Diagnostic Reasoning, and Refusing to Answer. The inter-rater reliability for the two rounds of error categorization yielded a kappa coefficient of 0.859. After further discussion, the raters reached a consensus on 77 initially disputed cases.

The LLMs with varying performance levels exhibited different patterns of errors (Fig. 4c and detailed in Supplementary Table 11). For GPT-3.5t and Gemini-1.0-pro, the most prevalent errors were Ignoring Key Clues (56.0% and 54.8%, respectively) and Knowledge Deficiency (19.3% and 32.3%, respectively). In contrast, higher-performing models such as Claude 3.5 Sonnet, Claude 3 Opus, and GPT-4o demonstrated almost no instances of Knowledge Deficiency (0%, 0%, and 4.2%, respectively). The predominant errors for the three advanced models were Misinterpretation of Key Clues (54.7%, 38.9%, and 18.8% for Claude 3.5 Sonnet, Claude 3 Opus, and GPT-4o, respectively) and Ignoring Key Clues (39.1%, 55.6% and 72.9% for Claude 3.5 Sonnet, Claude 3 Opus, and GPT-4o, respectively).

Five physicians who participated in the questionnaire survey analyzed their own diagnostic errors (Supplementary Table 12). The average coverage rate of these five physicians was 33.1% (95% CI: 28.3%–38.4%). Based on actual diagnoses and clinical interpretations, the physicians analyzed a total of 224 incorrect responses (Fig. 2e and Supplementary Table 13). The distribution of error categorization was as follows: Knowledge Deficiency (64.3%), Ignoring Key Clues (10.7%), Misinterpretation of Key Clues (15.6%), Inadequate Diagnostic Reasoning (8.0%), and Others (1.3%). Advanced LLMs such as Claude 3.5 Sonnet (0%, 95% CI: 0–5.8%), Claude 3 Opus (0%, 95% CI: 0–4.2%), and GPT-4o (4.2%, 95% CI: 1.5–10.4%) exhibited significantly lower rates of Knowledge Deficiency compared to the five physicians (64.3%, 95% CI: 57.7%–70.4%, *p* < 0.001).

In the subgroup analysis, the five physicians made 79 errors in GI cases and 145 errors in non-GI cases. The proportion of Knowledge Deficiency errors in non-GI cases was significantly higher than that in GI cases (72.4% vs. 49.4%, *p* < 0.001). In contrast, advanced LLMs such as Claude 3.5 Sonnet and Claude 3 Opus demonstrated no Knowledge Deficiency errors in both GI and non-GI cases.

## Discussion

This study first systematically explored human-machine comparisons between multiple widely used LLMs and 22 experienced gastroenterologists in an offline dataset. In the offline dataset comprising 67 challenging cases with primary GI symptoms, advanced LLMs such as Claude 3.5 Sonnet and GPT-4o significantly outperformed the gastroenterologists in diagnostic coverage rate. With the iteration and upgrade of LLMs, both the diagnostic coverage and accuracy of the new LLMs improved significantly. The study also included detailed analyses of hallucinations, diagnostic errors, diagnostic time, and diagnostic costs. The study highlights the potential of the advanced LLMs (Claude 3.5 Sonnet, Claude 3 Opus, and GPT-4o) to expand the diagnostic thinking of physicians and complement their experience limitations in diagnosing challenging cases.

Compared with previous studies^10,12,16,20–24^, our study utilized 67 closed-source and real-world challenging cases instead of online cases, exam questions, and fictional cases. This approach may reduce bias caused by information leakage when assessing the diagnostic abilities of LLMs and physicians^15,25,26^. Information leakage could potentially lead to bias that overestimates diagnostic performance. The study showed that physicians’ age, experience, and average question-answering duration positively correlated with accuracy or coverage rate. This suggests that the dataset can effectively capture variations in diagnostic performance among physicians with different experience levels and attentiveness. Meanwhile, previous studies frequently used multiple-choice questions or asked LLMs to provide a single diagnosis to evaluate the diagnostic capabilities of LLMs^10,17,20^. However, in real-world clinical workflow, physicians do not have explicit options and often need to consider a range of differential diagnoses. In our study, LLMs were asked to provide multiple diagnoses, and the evaluation of coverage aligns more closely with the actual needs of physicians. Recently, Ethan et al. found the diagnostic accuracy of the physicians using one kind of LLMs (GPT-4) did not surpass that of the physicians using traditional auxiliary methods in 6 challenging cases^17^. The limited sample size may constrain the generalizability of the findings. Moreover, the physicians in Ethan’s study were not experienced enough (median years in practice = 3). Although our study did not address human-AI collaboration, it offers several methodological strengths, including a broader range of LLMs, participation of more experienced clinicians, an expanded set of challenging cases, and comprehensive analyses of errors and hallucinations. Meanwhile, we not only explored the diagnostic accuracies of LLMs or physicians but also discussed the coverage differences between LLMs and physicians. We found advanced LLMs are expected to provide instructive diagnostic scope to physicians in diagnosing challenging cases, which is different from that of Ethan’s. This discrepancy may be related to the performance improvements of new LLMs, our choice of evaluation metrics, and the larger dataset. We believe that our research conclusions more accurately reflect the diagnostic value of rapidly developing LLMs for challenging cases at the current stage.

The study demonstrated that Claude 3.5 Sonnet achieved the highest performance in the tests, excelling in both coverage rate and accuracy. In the subgroup analysis, gastroenterologists achieved better performance in the GI subgroup than in the non-GI subgroup, whereas LLMs showed the opposite trend. This suggests that the gastroenterologists were less adept at diagnosing non-GI diseases that present with nonspecific GI symptoms. Despite the non-GI cases presenting with primary GI symptoms in the dataset being relatively straightforward, even senior gastroenterologists found them challenging to diagnose. Error analysis revealed that the proportion of Knowledge Deficiency in the non-GI subgroup was significantly higher than in the GI subgroup, further supporting this observation.

For the GI subgroup, the coverage rate of Claude 3.5 Sonnet and Claude 3 Opus was significantly higher than that of the 22 gastroenterologists, although their accuracy was only comparable to the upper level of GI specialists. This indicates that while LLMs may not yet surpass top specialists within their specific fields, they can offer valuable diagnostic scope to most specialists. For some challenging cases with primary GI symptoms, doctors were not sure whether their final diagnosis was a GI disease before confirmation. Therefore, the performance of LLMs in challenging cases with primary GI symptoms still needs to be comprehensively evaluated in the context of non-GI cases. For the non-GI subgroup, Claude 3.5 Sonnet, Claude 3 Opus, and GPT-4o can outperform all participating gastroenterologists. This aligns with real-world clinical workflows, where gastroenterologists tend to consult specialists from other disciplines for diagnostic advice.

Error analysis results indicate that, compared to human physicians’ diagnostic limitations attributable to knowledge gaps, Claude 3.5 Sonnet, Claude 3 Opus, and GPT-4o showed almost no knowledge deficiency errors. This reflects that advanced LLMs are expected to serve as “experts” across multiple disciplines, compensating for the knowledge limitations of physicians in diagnosing challenging cases. Moreover, our cost analysis demonstrates that, compared with costly MDT discussions, the expenses of LLMs are much lower. The use of LLMs to assist physicians in diagnosis may not significantly increase consultation fees. Additionally, LLMs demonstrated potential advantages over traditional auxiliary methods like search engines and literature databases, including higher coverage rates, time-saving benefits, and reduced reliance on the user’s clinical experience. LLMs have the potential to become new diagnostic support tools for physicians.

We also further evaluated the performance of LLMs under conditions of sparse clinical information that may be encountered in clinical practice. We conducted additional diagnostic tests using medical records that retained only admission notes and basic laboratory examinations (such as complete blood count and stool routine tests) at admission (Supplementary Note 2 and Supplementary Table 14). The coverage rates for Claude 3.5 Sonnet, GPT-4o, and Gemini-1.5-pro were 45.1%, 38.8%, and 33.6%, respectively. Despite a significant decrease in coverage rates for all LLMs (*p* < 0.05), Claude 3.5 Sonnet’s coverage rate of 45.1% still surpassed the average coverage rate of the 22 physicians (29.5%) on the original detailed dataset (*p* < 0.001). This reflects the robustness of Claude 3.5 Sonnet.

Furthermore, we supplemented our evaluation with publicly available cases from the NEJM over the past 5 years to assess LLMs’ performance across multidisciplinary cases (see Supplementary Note 3). When provided with admission notes only, Claude 3.5 Sonnet, GPT-4o, and Gemini-1.5-pro achieved coverage rates of 68.2%, 48.2%, and 50.4%, respectively (Supplementary Table 15). Notably, these LLMs demonstrated similar or higher diagnostic performance in cases with primary non-GI symptoms compared to those with primary GI-related symptoms in the NEJM dataset (Supplementary Table 16). This suggests that advanced LLMs can provide potentially valuable diagnostic scope across a broader spectrum of complex clinical scenarios. The performance of LLMs in scenarios with admission information suggests their potential in the decision-making of challenging cases such as triage, referrals, and multidisciplinary consultations.

Through a comparative analysis of new and old LLMs, we found that the new LLMs from Anthropic, OpenAI, and Google demonstrated significant performance improvements in both diagnostic coverage and accuracy compared to last year’s models. Hallucinations are considered one of the significant drawbacks of LLMs. We found that hallucinations often manifested as fabricated details introduced by the LLMs to substantiate their diagnoses. The hallucination analysis shows that while hallucinations were more frequent in LLMs with low performance, they did not necessarily lead to incorrect diagnoses. Additionally, our study did not perform multi-turn interactions with the LLMs. Each query was made independently without retaining conversation history. Consequently, further research is needed to determine whether hallucinations might influence subsequent responses in continuous dialogs. To our knowledge, little research has systematically analyzed the relationship between hallucinations and diagnostic errors. This intriguing finding warrants further investigation. Moreover, due to the inherent randomness of LLMs, their performance can fluctuate under identical conditions. The randomness of outputs is another feature of LLMs. When the temperature was set to 0, the responses from models like Claude 3.5 Sonnet, Gemini-1.5-pro, and Claude 3 Opus showed only moderate self-consistency across four rounds of testing, with moderate Krippendorff’s Alpha coefficients of 0.780, 0.720, and 0.697, respectively. However, the randomness in LLM-generated content also suggests that repeated queries may further improve coverage rates, which warrants further exploration. We believe that more advanced LLMs will be trained and offer better diagnostic performance, less hallucinations, and higher consistency.

Besides closed-source LLMs, there are also open-source LLMs fine-tuned on medical knowledge. For example, PULSE is a fine-tuned LLM with promising performance in certain tests (such as MedQA-USMLE and MedicineQA)^27^. PULSE was also tested using our dataset. We found that its performance was inferior to that of advanced closed-source LLMs (see Supplementary Note 4). This discrepancy may be attributed to the performance of PULSE’s pre-trained model and the composition of its fine-tuning dataset. Moreover, the deployment and use of fine-tuned LLMs often require more computational resources and specialized technical expertise, making them less accessible than online-available LLMs.

The study only used text-based clinical information to evaluate the diagnostic performance of the LLMs in challenging cases. More comprehensive clinical data, such as medical images, were not involved in the study. With the development of multimodal large language models (MLLMs), these models have shown considerable performance in handling image and audio tasks^28^. Among these models, GPT-4V has been widely used to evaluate its ability to recognize medical images. Recent studies have assessed GPT-4V’s capabilities in medical imaging tasks with mixed results^29–32^. For instance, Dehdab et al. conducted a retrospective study to evaluate GPT-4V’s performance in diagnosing chest CT scans^29^. GPT-4V achieved an overall diagnostic accuracy of 56.8%. Specifically, for non-small cell lung cancer, the sensitivity was 27.3% and specificity was 60.5%; for COVID-19, sensitivity was 13.6% and specificity was 64.3%; and for control cases, sensitivity was 31.8% with a specificity of 95.2%. Similarly, AlRyalat et al. assessed GPT-4V’s ability to recognize glaucoma using 400 test fundus images^30^. GPT-4V achieved 94.44% specificity but had a lower sensitivity of 50%. In our study, due to the unavailability of complete medical imaging data for the offline dataset, we were unable to evaluate multimodal performance. However, based on current research findings, we believe MLLMs still have significant potential in interpreting radiological images for common diseases. Given that challenging cases occur less frequently than common diseases, we speculate that the performance of MLLMs on challenging cases might further decline. Moreover, some researchers have reported that integrating medical images into patient records does not enhance the performance of GPT-4V compared to using text descriptions alone^33,34^. MLLMs may still require more training on high-quality and diverse clinical data. We believe that while their current performance remains inadequate, MLLMs represent an important direction for future development. Further investigation is also needed to explore whether other MLLMs’ multimodal capabilities offer advantages over text-based diagnoses in challenging cases.

Undiagnosed diseases are more prone to misdiagnosis, unnecessary invasive procedures, and other adverse events^1,2^. Utilizing LLMs to assist physicians in improving diagnostic accuracy in challenging cases is a potential approach to ameliorate this situation. However, how to better and more safely harness LLMs for accurate diagnosis remains an important issue. Although LLMs have shown potential in diagnosing challenging cases, the clinical application of LLMs faces challenges such as hallucinations, harmful information, and racial and gender biases, which can negatively impact diagnostic outcomes. For instance, a study showed that in 44 cases of infected patients, 16% of the infection treatment plans provided by GPT-4 were harmful^35^. Therefore, physicians need to avoid being misled by erroneous information from LLMs. Diagnostic opinions generated by LLMs should undergo rigorous evaluation by human experts. More clinical trials are urgently needed to assess misdiagnoses and other adverse events caused by LLMs. Furthermore, guidelines, safety protocols, accountability frameworks, and legal regulations should be established. LLM technology companies also need to pay attention to issues of racial and gender biases to prevent related adverse events and medical injustice. Additionally, the regional availability of advanced LLMs may also exacerbate the imbalance in medical resources in the future. Moreover, privacy breaches and ethical and legal risks are significant factors limiting the clinical application of LLMs^10,36–40^. Effectively protecting private information when LLMs process medical data is a crucial prerequisite for their clinical utilization. Considering that localized deployment of LLMs requires enormous computing resources and technical expertise, we recommend that medical centers, LLM service providers, and regulators jointly develop privacy protection mechanisms to reduce ethical and legal risks. Additionally, LLMs require structured and complete medical information to evaluate challenging cases. In this study, we provided structured text information to LLMs. However, the lack of effective tools to integrate fragmented data (such as radiological and laboratory examinations) presents significant challenges to the clinical application of LLMs. This requires medical centers to further digitize medical information and break down the barriers between different medical information systems. We believe that collaboration among hospitals, technology companies, and policymakers is essential to make LLMs better assist physicians in providing improved healthcare services for undiagnosed cases.

Our study has limitations. The primary limitation of this study was that retrospective cases may not fully reflect actual clinical scenarios. Although physicians were allowed to consult colleagues in the study, they were surveyed individually. The datasets were well-organized, but medical records such as test results and radiological reports in the real-world are multimodal, fragmented, and unordered. Therefore, as mentioned above, effective tools to integrate these fragmented data are needed for prospective studies. Second, due to the scarcity of experienced physicians and the time-consuming nature of diagnosing challenging cases, this study only evaluated 22 gastroenterologists across 67 challenging cases involving primary GI symptoms. Further comparison is needed to assess the differences in diagnostic capabilities between LLMs and physicians across a broader range of medical specialties in challenging cases. Our testing of several LLMs on the NEJM challenging cases can provide supplementary evidence. LLMs showed similar or higher coverage rates and accuracies in NEJM cases with primary non-GI symptoms (Supplementary Note 3). This may reflect the potential value and robustness of LLMs in diagnosing a wider range of challenging cases. Third, this study did not include an experimental group to investigate the interaction effects between humans and LLMs. New randomized controlled trials would help further explore the potential advantages of LLMs compared to traditional auxiliary methods. Fourth, the study only used simple prompts to ask LLMs for diagnoses. The diagnostic potential of LLMs with post-training technology warrants further exploration^41–45^. We are currently conducting new research to further improve LLMs’ performance in challenging cases.

In conclusion, advanced LLMs, particularly Claude 3.5 Sonnet, demonstrated superior diagnostic performance compared to the most experienced gastroenterologists in challenging cases with primary GI symptoms. As a novel assistive tool, advanced LLMs are expected to provide instructive diagnostic scopes for challenging multidisciplinary cases.

## Methods

### Dataset construction

The dataset of diagnostically challenging cases was collected from Chinese printed medical case books edited by national tertiary hospitals. The inclusion and exclusion criteria are as follows: (1) cases must have primary GI symptoms and diagnostic difficulties; (2) the final diagnosis must account for the GI symptoms and other symptoms and examination results; (3) the diagnosis should be attainable without further invasive procedures (such as surgery); (4) the case records should not be available online.

Two panels were involved in dataset construction and diagnostic conclusion evaluation, respectively (Supplementary Table 17). Panel A consisted of 6 gastroenterologists and 1 pathologist with a median (IQR) of 16 (10.5–17.5) years of clinical experience. Panel A selected and enrolled cases by the criteria. Panel B consisted of 3 senior gastroenterologists. Each had over 20 years of GI clinical experience and more than 15 years of experience in writing medical English literature. Panel B participated in the dataset collection and evaluation of the diagnostic test. All panel members were affiliated with national tertiary hospitals.

Case records were digitized and refined by eliminating descriptions that directly revealed final diagnoses. Medical imaging findings were presented in text format rather than as images. The integrity of the information necessary for correct diagnostic reasoning was maintained. Reference diagnoses were based on the reported diagnoses. As the gastroenterologists participating in the survey are native Chinese speakers, the Chinese version of the medical records was used to evaluate the performance of human gastroenterologists. The initial English draft of the records was translated using DeepL Translate (https://www.deepl.com/). This draft underwent two rounds of error-checking through Chinese-English comparison using GPT-4. Then, two medical doctors in Panel A individually reviewed and revised all translations. Each doctor had at least 13 years of clinical and medical writing experience. The two medical doctors compared the translated content and merged their revisions. Finally, the records were revised by a medical professor from Panel B with over 21 years of professional experience in medical writing and clinical practice. The finalized English version of the records was used for testing LLMs.

The study was approved by the Xijing Hospital Ethics Committee (KY20242031-C-1) and followed the Declaration of Helsinki principles. As de-identified medical cases were used, informed consent was not required.

### LLMs and physicians

In this study, seven widely used LLMs were investigated for their diagnostic capabilities, including GPT-3.5t, GPT-4o, Gemini-1.0-pro, Gemini-1.5-pro, Claude-2.1, Claude 3 Opus and Claude 3.5 Sonnet. The dates of querying and API details are shown in Supplementary Table 18.

Thirty gastroenterologists from 17 tertiary hospitals, each with more than 10 years of clinical experience in GI participated in this survey (Supplementary Table 12). These physicians were not involved in the processing of the dataset and were blinded to its content.

### Study design

The investigation consists of two components: querying LLMs and conducting surveys with physicians (Fig. 1). Each case from the English version dataset was queried individually for each LLM. The LLMs were asked to provide several possible diagnoses and one most likely diagnosis (Fig. 2). The temperature parameter of the LLMs was set to 0. All LLMs were accessed via APIs, with no retention of previous conversation history. Each LLM underwent four rounds of repeat queries. If an LLM declined to answer, the query was resubmitted up to two more times. The processing time and associated API costs for each query were documented.

For the physician component, the Chinese version dataset was randomly divided into multiple online questionnaires, each containing up to 10 diagnostic questions. Questionnaires were distributed daily, with a two-week completion window. Participants were also asked to provide possible diagnoses and the most likely diagnosis. Apart from LLMs, physicians were allowed to use auxiliary methods such as search engines, academic databases, and consulting colleagues (Table 2). The auxiliary methods used for each case’s diagnostic reasoning and the time spent on each questionnaire were recorded. Physicians were excluded if their response rate to diagnostic questions was less than 40.0%, which was considered insufficient for the evaluation of diagnostic capability.

**Fig. 5:**
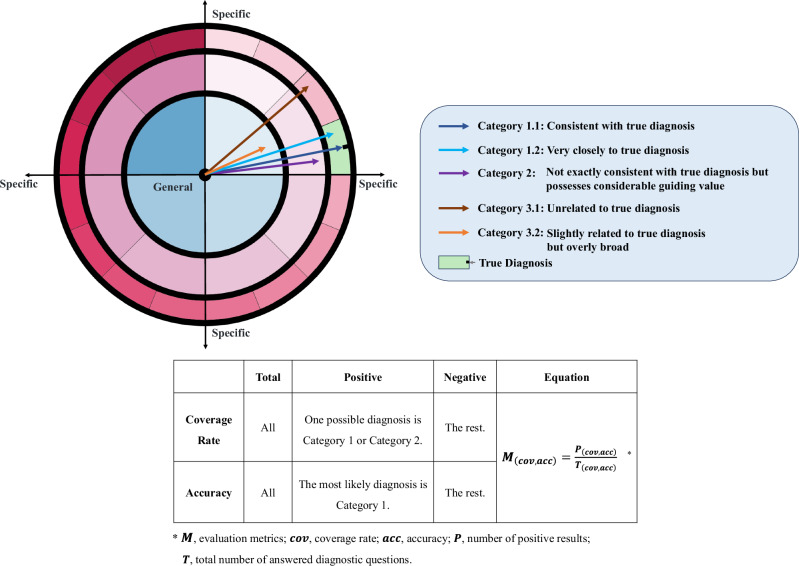
Criteria for determining the correctness of diagnostic outcomes and the calculation of coverage rate and accuracy. The dartboard graph shows the disease spectrum. Zones with different colors represent different diseases. The closer to the bullseye, the more general the disease diagnosis; the closer to the edge, the more specific the diagnosis. The different wedge-shaped areas at the same angle represent diagnoses of varying specificity within the same broad disease category. The arrows point to regions that represent different diagnostic conclusions. These conclusions are divided into three categories based on the evaluation in Panel B. Subsequently, the corresponding coverage and accuracy are calculated using the equation in the table.

The criteria for the assessment of diagnostic conclusions and the calculation of measures are detailed in Fig. 5 and Supplementary Note 5. The conclusions were categorized into three groups. Category 1 diagnoses are accurate diagnoses (Category 1.1) or very close to final diagnoses (Category 1.2). Category 2 represents conclusions that are not exactly consistent with the final diagnoses but possess considerable guiding value, and Category 3 represents incorrect (Category 3.1) or overly broad diagnoses (Category 3.2). Based on the rules, Panel B discussed and categorized all the diagnostic conclusions. The specific criteria for categorizing responses to each question can be found in Supplementary Table 3. The primary outcome measure was the diagnostic coverage rate, defined as the proportion of cases with diagnostic conclusions falling into Category 1 or Category 2. The secondary outcome was diagnostic accuracy, representing the proportion of cases where the most likely diagnoses were classified into Category 1. Other metrics included the time spent per case, the average number of possible diagnoses per case, and the average fee of different LLMs per query.

### Hallucination analysis method

In the study, hallucinations are defined as any fabricated information in the LLMs’ responses, using the medical records as a reference. Such information includes fabricated symptoms, examinations, medication history, and so on. The hallucination analysis was conducted by four physicians from Panel A, divided into two independent groups. Both groups carefully compared all responses by the LLMs against the original medical records. To ensure reliability, the results from both groups underwent a consistency analysis to evaluate inter-rater agreement. For contentious issues, two additional physicians from Panel A discussed and reached a consensus decision. Further analysis was conducted to examine the correlation between hallucinations and incorrect answers. Additionally, the correlation between varying diagnostic performance of LLMs and the frequency of hallucinations was investigated.

### Error analysis method

The error analysis of the LLMs was conducted by two physicians from Panel B through a four-step process (Supplementary Note 6). The causes of errors were categorized into five types: (1) Refusing to Answer, (2) Knowledge Deficiency, (3) Ignoring Key Clues, (4) Misinterpretation of Key Clues, and (5) Inadequate Diagnostic Reasoning. Upon completion of the analysis, an inter-rater reliability assessment was performed on their results. Any discrepancies in classification were resolved through discussion between the two raters until a consensus was reached.

The error analysis of the physicians’ responses was conducted by the physicians themselves. Complete medical records containing true diagnoses were provided. The types of errors identified are similar to those of the LLMs and include five categories: (1) Knowledge Deficiency, (2) Ignoring Key Clues, (3) Misinterpretation of Key Clues, (4) Inadequate Diagnostic Reasoning, and (5) Others. Depending on the circumstances, Knowledge Deficiency was further classified into four specific situations (detailed in Supplementary Note 7). Physicians were asked to classify their errors into one category.

### Statistical analysis

The sample size of the challenging case dataset was estimated using PASS 2021 (NCSS, LLC. Kaysville, Utah, USA) (Supplementary Note 8). Shapiro–Wilk test was used to evaluate data normality. McNemar’s test, Chi-square test, and Fisher’s exact test were used to analyze the significance of differences in accuracies and coverage rates between LLMs and physicians. The consistency of LLM responses was analyzed using Krippendorff’s Alpha (Supplementary Note 9). The inter-rater reliability for the two rounds of hallucination analysis and error categorization was evaluated using Cohen’s kappa. Wilson’s method was used to calculate 95% CIs. Pearson correlation, point-biserial correlation, and Phi correlation were calculated to explore variables correlated with coverage rate or accuracy, such as the age and clinical experience of the physicians, and occurrences of hallucinations. The odds ratio was calculated to evaluate the strength and direction of the association between hallucinations and error responses from LLMs.

All statistical analyses were performed using SciPy and sci-kit-learn in Python version 3.9, adhering to a two-sided significance threshold of 0.05.

## Supplementary information

Supplemental Material

## Data Availability

The challenging cases used in this study are not publicly available due to their integration within a proprietary research framework, which ensures the feasibility of ongoing and future studies. Readers can access these cases through the International Standard Book Numbers (ISBNs) or Digital Object Identifiers (DOIs) provided in the Supplementary Tables 1 and 2. The responses of the LLMs or derived data are available from the corresponding author.

## Code availability

The code for the PULSE-20b model is publicly available at https://github.com/openmedlab/PULSE.
