# Supplementary material for: Multiple large language models versus experienced physicians in diagnosing challenging cases with gastrointestinal symptoms
Source: NPJ Digit Med. 2025 Feb 5;8:85. doi: 10.1038/s41746-025-01486-5 (PMC11799458; doi:10.1038/s41746-025-01486-5)
Supplement: Supplementary file 1 — Supplemental Material [file 41746_2025_1486_MOESM1_ESM.pdf]

Supplemental Material List

Supplementary Note

- Supplementary Note 1. Multiple linear regression for predicting the time consumption per questionnaire
- Supplementary Note 2. Performance of LLMs in cases of sparse clinical Information
- Supplementary Note 3. Collection of the NEJM dataset and testing of the LLMs
- Supplementary Note 4. Testing of an open-source fine-tuned LLM (PULSE)
- Supplementary Note 5. Criteria for categorization of diagnostic outcomes
- Supplementary Note 6. Error analysis of the LLMs' responses
- Supplementary Note 7. Error analysis of the physicians' answers
- Supplementary Note 8. Sample size evaluation
- Supplementary Note 9. Answer consistency of LLMs in four rounds of queries

Supplementary Table

- Supplementary Table 1. Cases presenting with primary gastrointestinal symptoms in the Case Records of the Massachusetts General Hospital, published in the New England Journal of Medicine
- Supplementary Table 2. International standard book numbers of the chosen medical case books
- Supplementary Table 3. Dataset of challenging cases
- Supplementary Table 4. Diagnostic performance evaluation of physicians
- Supplementary Table 5. Correlation analysis between diagnostic coverage rate/accuracy and other variables
- Supplementary Table 6. Correlation analysis between token numbers and judgment classification
- Supplementary Table 7. Diagnostic performance evaluation of LLMs in GI and non-GI cases
- Supplementary Table 8. Time and cost for various LLMs
- Supplementary Table 9. Occurrence of hallucinations in LLM responses across four test rounds
- Supplementary Table 10. Analysis of the correlation and odds ratio between hallucinations in LLM responses and erroneous responses
- Supplementary Table 11. Error categorization of LLMs' responses
- Supplementary Table 12. Baseline of the gastroenterologists attending questionnaire investigation
- Supplementary Table 13. Error categorization of physicians' responses
- Supplementary Table 14. Diagnostic performance of LLMs when only providing admission information and routine laboratory test results
- Supplementary Table 15. Performance of five LLMs on the NEJM dataset
- Supplementary Table 16. Performance of five LLMs on the NEJM dataset patients with primary GI and Non-GI symptoms
- Supplementary Table 17. Baseline of the members in Panel A and Panel B
- Supplementary Table 18. Model information and query date of the involved LLMs
- Supplementary Table 19. The coverage rate, accuracy, and consistency evaluation of PULSE

Supplementary Figure

- Supplementary Figure 1. Diagnostic performance, statistical significance, and consistency analysis of 7 different LLMs across four query rounds

- Supplementary Figure 2. Statistical significance of the coverage rate and the accuracy between the LLMs in each round and the physicians
- Supplementary Figure 3. Statistical significance of the coverage rate and the accuracy between different LLMs in the GI and non-GI case subgroups
- Supplementary Figure 4. The comparison of PULSE with other closed-source LLMs or the performance of the physicians

Supplementary Note

Supplementary Note 1. Multiple linear regression for predicting the time consumption per questionnaire

Physicians spent an average of 6.60 minutes per question. To assess the answering time of participating physicians when using varying numbers of auxiliary methods for diagnosis, this study developed a multiple linear regression model. The total response time for each questionnaire and the number of auxiliary methods employed for each question were known. The count of questions using 0 to 3 auxiliary methods served as independent variables, while the corresponding questionnaire response time was the dependent variable for multiple linear regression modeling. A total of 113 complete questionnaires, each recording the assistive tools used, were incorporated into the multiple linear regression model. The z-score method was applied to eliminate outliers on both ends, preserving 90% of the effective values. Multiple linear regression was conducted, resulting in Equation (1).

$$T(min) = \sum_{\{i=0\}}^{3} a_i x_i \quad (1)$$

In Equation (1), a0 = 3.93, a1 = 8.74, a2 =11.76, a3 = 17.94. The coefficient of determination (R2) is 0.46, and the mean squared error (MSE) is 603.1. T represents the predicted time spent on a questionnaire, x0 represents the number of questions answered without any auxiliary method, and x1, x2, and x3 represent the number of questions answered using one, two, and three auxiliary methods, respectively. The predicted time spent on one case without auxiliary methods, and with one, two, and three auxiliary methods, was 3.93, 8.74, 11.76, and 17.94 minutes, respectively. It should be noted that these estimates were based on the average effect of the number of methods rather than specific types, and individual auxiliary methods may vary in time requirements. Therefore, if only one auxiliary method was used, it is predicted to take approximately 4.81 minutes more than using no auxiliary method. Considering the average time per query to large language models (LLMs, 0.19 minutes), using LLMs for diagnosis saves more than 90% of the additional query time compared to using one traditional auxiliary method.

Supplementary Note 2. Performance of LLMs in cases of sparse clinical information

Compared to the closed-source dataset used in this study, real-world challenging cases often provide sparser clinical information and may lack certain diagnostic indicators. To evaluate the performance of LLMs under these conditions, we retained admission records along with some routine basic test results (such as complete blood count, routine urinalysis, and routine stool tests). We tested GPT-4o, Gemini-1.5-pro, and Claude 3.5 Sonnet. The testing and evaluation methods were consistent with those used for the original proprietary dataset, as described in the main text. The results of the four rounds of testing are shown in Supplementary Table 14. The performance of these LLMs on the sparse dataset significantly declined compared to their performance on the original dataset ($p < 0.05$ for all comparisons). However, the performance ranking among the models remained similar to that observed with the original dataset. Notably, Claude 3.5 Sonnet demonstrated significantly higher coverage in the sparse clinical information dataset compared to the coverage provided by 22 physicians on the original dataset (45.1% vs. 29.5%, $p < 0.001$). This suggests that advanced LLMs may still offer valuable diagnostic insights, even when faced with sparse clinical information.

Supplementary Note 3. Collection of the NEJM dataset and testing of the LLMs

We retrieved 204 papers from the "Case Records of the Massachusetts General Hospital" published in NEJM between May 2019 and May 2024. After a review by Panel B, 34 non-challenging cases, such as some non-challenging COVID-19 cases and those involving innovative technologies, were excluded. This resulted in 170 challenging case reports. The information from the "Presentation of Case" sections was organized to serve as challenging case questions for testing the performance of LLMs. Image information was replaced with textual descriptions of the legends.

This dataset was used to conduct four rounds of repeated testing on GPT-3.5t, GPT-4o, Gemini-1.5-pro, Claude 3 Opus, and Claude 3.5 Sonnet. The prompt words, access methods, and evaluation methods were the same as those described in the main text. The test results are shown in Supplementary Table 15 and 16. Regardless of whether the chief complaint involved suspected GI symptoms or non-GI symptoms, GPT-4o, Gemini-1.5-pro, Claude 3 Opus, and Claude 3.5 Sonnet demonstrated notable performance. The diagnostic performance in the non-GI subgroup was similar to or slightly higher than that in the GI subgroup. This suggests that LLMs have potential value in diagnosing a broader range of challenging cases beyond those primarily involving the GI system.

Supplementary Note 4. Testing of an open-source fine-tuned LLM (PULSE)

The PULSE model was further fine-tuned using approximately 4,000,000 Chinese medical and general domain instruction data points [1]. It demonstrated excellent performance on multiple medical question-answering datasets, including MedQA-USMLE and MedicineQA. We deployed the PULSE-20b model on a server with dual A100 (40GB) GPUs. The model's computational precision was set to fp16. The initial system prompt was removed, allowing the model to provide diagnoses based on the cases we provided. The specific prompt setup method was the same as described in the main text. Since this model was fine-tuned on medical question-answer pairs in Chinese, all test cases were queried in Chinese. After four rounds of testing, we evaluated the responses using the same criteria as in the main text. PULSE-20b consistently refused to answer Case 45 and Case 52 in all four rounds. We repeated the test four more times, and it still refused to answer. Upon detailed analysis of these two cases, we found no content that violated legal or ethical standards. The performance of PULSE-20b is shown in Supplementary Table 7 and 19. Although its consistency was excellent (Krippendorff's Alpha = 1.000), its coverage and accuracy were lower than those of other LLMs ($p < 0.005$, see Supplementary Figure 4). After error analysis, we found that the main reason for PULSE-20b's underperformance was Knowledge Deficiency (Supplementary Table 11 and Supplementary Figure 4).

Supplementary Note 5. Criteria for categorization of diagnostic outcomes

The diagnostic conclusions provided by LLMs and physicians followed the same judgment criteria. The diagnostic conclusions were divided into 3 categories as follows (Figure 5):

Category 1: Being consistent with the true diagnosis or possessing significant guiding value

1.1 If the provided diagnostic conclusion is consistent with the true diagnosis, it is considered correct and classified as Category 1.

1.2 If the provided diagnostic conclusion is not exactly consistent with the true diagnosis but very close to the true diagnosis, it is classified as Category 1. For example, lymphoma is classified as Category 1 for Case 42 (true diagnosis: small intestinal T-cell lymphoma).

Category 2: Possessing considerable guiding value

If the provided diagnostic conclusion is not exactly consistent with the true diagnosis but can point towards the true diagnosis and has considerable guiding value, it is classified as Category 2. For example, rheumatoid arthritis is classified as Category 2 for Case 45 (true diagnosis: Felty's syndrome).

Category 3: Being incorrect or lacking guiding value

3.1 If the provided diagnostic conclusion is unrelated to the true diagnosis and fails to guide the diagnostic process toward the correct final diagnosis, it is considered incorrect or lacking diagnostic value. In such cases, the diagnosis is classified as Category 3, indicating that it does not contribute meaningfully to reaching an accurate diagnosis.

3.2 If the provided diagnostic conclusion has a slight relation to the true diagnosis but is overly broad and lacks clear guiding value for the diagnosis, it is classified as Category 3. For example, achalasia is considered wrong for Case 10 (true diagnosis: Jackhammer esophagus). Gastric cancer is classified as Category 3 for Case 23 (true diagnosis: Hepatoid adenocarcinoma of the stomach).

Supplementary Note 6. Error analysis of the LLMs' responses

The error analysis of LLMs was conducted through a four-step process, categorizing the causes of errors into five types: (1) Refusing to Answer, (2) Knowledge Deficiency, (3) Ignoring Key Clues, (4) Misinterpretation of Key Clues, and (5) Inadequate Diagnostic Reasoning. The process is as follows:

Step 1: Errors were classified as Refusing to Answer when the LLM declined to respond twice.
Step 2: A three-round dialogue was conducted to assess whether the LLM fully understood the correct diagnostic methods for the disease in question:

First round: The LLM was asked about the diagnostic criteria for the correct diagnosis, including symptoms, laboratory indicators, imaging features, histological characteristics, genetic abnormalities, and molecular features (if applicable).
Second round: The LLM was prompted to provide additional information.
Third round: The LLM was asked to reflect on any potentially overlooked diagnostic points.
Human physicians analyzed the results of these three rounds of dialogue. If the LLM demonstrated deficiencies in its understanding of the disease's diagnosis, the error was attributed to Knowledge Deficiency.

Step 3: For erroneous diagnoses not classified as Knowledge Deficiency, the LLM's generated reasoning process was examined to determine if crucial diagnostic clues had been overlooked. If key clues were missed, the error was categorized as Ignoring Key Clues.
Step 4: The remaining unclassified errors were further evaluated to assess whether the interpretation of key diagnostic clues was accurate. If the interpretation was incorrect, the error was classified as Misinterpretation of Key Clues. If the interpretation was correct, but the LLM diagnosed a different disease or multiple unrelated diseases, the error was categorized as Inadequate Diagnostic Reasoning. This category typically included cases involving syndromes with multi-system symptoms, instances requiring the consideration of congenital diseases based on the patient's age and age of onset, or cases necessitating complex reasoning to explain atypical laboratory results.

The error analysis of the LLMs' responses was conducted by two physicians from Panel B. Upon completion of the analysis, an inter-rater reliability assessment was performed on their results. Any discrepancies in classification were resolved through discussion between the two raters until a consensus was reached.

Supplementary Note 7. Error analysis of the physicians' answers

The error analysis of the physicians' answers was conducted by the physicians themselves. They were provided with complete medical records containing the true diagnoses. Physicians were asked to classify their errors into one category. The types of errors identified were similar to those of the LLMs and included five categories: (1) Knowledge Deficiency, (2) Ignoring Key Clues, (3) Misinterpretation of Key Clues, (4) Inadequate Diagnostic Reasoning, and (5) Others.

The definitions of each type of error were as follows:

(1) Knowledge Deficiency:
   a. Uncertainty regarding the significance of certain laboratory indicators or imaging findings.
   b. Difficulty in determining whether laboratory indicators were abnormal.
   c. Complex medical conditions that involve multiple systems, requiring evaluation by a multidisciplinary team.
   d. Rare medical conditions that the physician was unfamiliar with.

(2) Ignoring Key Clues: The physician knew the diagnostic methods for the correct disease but overlooked crucial diagnostic clues during the diagnostic process.

(3) Misinterpretation of Key Clues: The physician was aware of the diagnostic methods for the correct disease and analyzed the key diagnostic clues; however, there was an error in interpreting these clues.

(4) Inadequate Diagnostic Reasoning: The physician knew the diagnostic methods for the correct disease and analyzed the key diagnostic clues correctly, but arrived at an incorrect conclusion in their reasoning, or used multiple unrelated diseases to explain the patient's complex condition.

Supplementary Note 8. Sample size evaluation

The sample size for the challenging medical cases dataset was determined using PASS 2021 (NCSS, LLC. Kaysville, Utah, USA). Based on the experience of Panel A in processing diagnostically challenging cases, the coverage rates of LLMs and physicians were estimated to be 60% and 40%, respectively. Verify the statistical parameters to ensure accuracy. If correct, consider explaining why these values were chosen. The estimated sample size was determined to be at least 67 cases.

Supplementary Note 9. Answer consistency of LLMs in four rounds of queries

Cases with the most likely diagnosis classified as Category 1 are assigned a value of 1. Cases with one possible diagnosis classified as Category 2, or where a possible (but not the most likely) diagnosis is classified as Category 1, are assigned a value of 2. Diagnostic conclusions classified as Category 3 are assigned a value of 0. Then, Krippendorff's Alpha was calculated to evaluate the consistency of the responses in the four rounds of queries.

Supplementary Table 1. Case presenting with primary gastrointestinal symptoms in the Case Records of the Massachusetts General Hospital, published in the New England Journal of Medicine *

DOI	Primary Symptom	Final Diagnosis
10.106/NEJMcpc1900142	A 39-Year-Old Woman with Palpitations, Abdominal Pain, and Vomiting	Hyperthyroidism due to Graves' disease.
10.1056/NEJMcpc1900594	A 38-Year-Old Woman with Abdominal Pain and Fever	Intestinal tuberculosis
10.1056/NEJMcpc1900596	A 41-Year-Old Pregnant Woman with Abdominal Pain	Acute suppurative appendicitis and peri-appendicitis.
10.1056/NEJMcpc1904039	A 48-Year-Old Man with Lymphoma and Abdominal Pain	Disseminated cryptococcosis.
10.1056/NEJMcpc1904049	A 14-Month-Old Boy with Vomiting	Congenital esophageal stenosis with fibromuscular thickening of the esophagus
10.1056/NEJMcpc1909625	A 34-Year-Old Man with Dyspnea, Odynophagia, and Abdominal Pain	Kaposi's sarcoma of the gastrointestinal tract.
10.1056/NEJMcpc1913469	An 11-Year-Old Boy with Vomiting and Weight Loss	Autoimmune primary adrenal insufficiency and celiac disease
10.1056/NEJMcpc1913473	A 44-Year-Old Man with Weight Loss, Diarrhea, and Abdominal Pain	Strongyloidiasis with human T-lymphotropic virus type 1 infection.
10.1056/NEJMcpc1913476	An 89-Year-Old Man with Recurrent Abdominal Pain and Bloody Stools	Ulcerative colitis.
10.1056/NEJMcpc1916257	A 55-Year-Old Man with Abdominal Pain, Joint Swelling, and	Pancreatitis, panniculitis, and polyarthritis syndrome.

DOI	Primary Symptom	Final Diagnosis
	Skin Lesions	
10.1056/NEJMcpc2027091	A 34-Year-Old Woman with Abdominal Distention and Acute Kidney Injury	Plasma cell myeloma (multiple myeloma)
10.1056/NEJMcpc2027094	A 16-Year-Old Boy with Headache, Abdominal Pain, and Hypertension	Pheochromocytoma.
10.1056/NEJMcpc2100274	A 76-Year-Old Woman with Nausea, Diarrhea, and Acute Kidney Failure	Lactic acidosis associated with metformin use.
10.1056/NEJMcpc2100278	A 37-Year-Old Woman with Abdominal Pain and Aortic Dilatation	Staphylococcus aureus bacteremia and infection of a vascular graft.
10.1056/NEJMcpc2103461	A 41-Year-Old Woman with Bloody Stools and Thrombocytopenia	Cytomegalovirus-induced immune thrombocytopenia.
10.1056/NEJMcpc2107344	A 33-Year-Old Pregnant Woman with Fever, Abdominal Pain, and Headache	Listeria monocytogenes bacteremia resulting in loss of fetus.
10.1056/NEJMcpc2107354	A 76-Year-Old Woman with Abdominal Pain, Weight Loss, and Memory Impairment	Lead poisoning.
10.1056/NEJMcpc2107356	A 50-Year-Old Woman with Pain in the Left Upper Quadrant and Hypoxemia	Pneumocystis jirovecii pneumonia.

DOI	Primary Symptom	Final Diagnosis
10.1056/NEJMcpc2115846	A 56-Year-Old Woman with Fever, Myalgias, Diarrhea, and Cough	Anaplasmosis
10.1056/NEJMcpc2115850	A 14-Year-Old Boy with Fever, Joint Pain, and Abdominal Cramping	Inflammatory bowel disease (Crohn's disease)
10.1056/NEJMcpc2115856	A 57-Year-Old Man with Chylous Ascites	High-grade B-cell lymphoma, not otherwise specified.
10.1056/NEJMcpc2201236	A 33-Year-Old Man with Chronic Diarrhea and Autoimmune Enteropathy	Immune dysregulation, polyendocrinopathy, enteropathy, X-linked (IPEX) syndrome.
10.1056/NEJMcpc2201239	A 72-Year-Old Man with Heartburn, Nausea, and Inability to Eat	Linitis plastica (invasive gastric adenocarcinoma)
10.1056/NEJMcpc2201248	A 58-Year-Old Woman with Fatigue, Abdominal Bloating, and Eosinophilia	Mansonella perstans infection.
10.1056/NEJMcpc2201249	A 56-Year-Old Man with Abnormal Results on Liver Testing	Primary biliary cholangitis with portopulmonary hypertension.
10.1056/NEJMcpc2211363	A 31-Year-Old Woman with Postpartum Abdominal Pain and Fever	Hepatic adenoma, inflammatory type, with associated ischemic necrosis and extensive hemorrhage
10.1056/NEJMcpc2211367	A 16-Year-Old Girl with Abdominal Pain and Bloody Diarrhea	Catastrophic antiphospholipid syndrome
10.1056/NEJMcpc2300896	A 25-Year-Old Woman with Abdominal Pain and Jerking Movements	Factitious disorder.

DOI	Primary Symptom	Final Diagnosis
10.1056/NEJMcpc2300903	A 53-Year-Old Woman with Celiac Disease and Upper Gastrointestinal Bleeding	Turner syndrome.
10.1056/NEJMcpc2301032	A 43-Year-Old Woman with Chronic Diarrhea, Hair Loss, and Nail and Skin Changes	Cronkhite–Canada syndrome.
10.1056/NEJMcpc2309725	An 8-Week-Old Male Infant with Inconsolable Crying and Weakness	Infant botulism

* The search time range for case reports is from May 2019 to May 2024.

Supplementary Table 2. International standard book numbers of the chosen medical case books

Number of Medical Case Books	International Standard Book Number
**1**	978-7-117-13071-4
**2**	978-7-5189-4773-7
**3**	978-7-117-16571-6
**4**	978-7-5679-1513-8
**5**	978-7-81072-882-9
**6**	978-7-03-061629-6
**7**	978-7-5439-8460-8
**8**	978-7-5641-9312-6
**9**	978-7-5377-2957-4
**10**	978-7-8113-6080-6
**11**	978-7-2290-4120-5

Supplementary Table 3. Dataset of challenging cases

Case ID	Disease Grouping	Disease Type	Token Number *	Diagnosis Categorization Criteria		
				Category 1.1	Category 1.2	Category 2
1	GI	Systemic scleroderma	454	Systemic sclerosis/Scleroderma/ Scleroderma with gastroesophageal involvement/ Systemic sclerosis (Limited cutaneous type - CREST Syndrome)	-	-
2	GI	Ménétrier's disease	425	Menetrier's disease	Hypertrophic gastropathy/ Hypertrophic gastritis	
3	GI	Obscure source of bleeding: Pancreatic duct stones causing bleeding	785	Pancreatic duct stones	-	Chronic pancreatitis/ Chronic Pancreatitis Complications/ Hemosuccus pancreaticus
4	GI	Congenital esophageal stenosis	323	Congenital esophageal stenosis		
5	GI	Autoimmune enteropathy	762	Autoimmune enteropathy		
6	GI	Very early-onset inflammatory bowel disease (IL-10RA gene deficiency)	619	Very early onset Crohn's disease/ IL-10 Receptor Deficiency		
7	GI	Cronkhite-Canada syndrome	703	Cronkhite-Canada syndrome (CCS)		
8	GI	Hepatic myelopathy	439	Hepatic myelopathy		
9	GI	Abdominal cocoon syndrome	380	Abdominal cocoon/ sclerosing encapsulating peritonitis/ Encapsulating Peritoneal Sclerosis		
10	GI	Jackhammer esophagus	309	Jackhammer esophagus	Hypercontractile esophagus	

11	GI	Cronkhite-Canada syndrome	547	Cronkhite-Canada syndrome		
12	GI	Glycogen storage disease	382	Glycogen storage disease/ Von Gierke disease		
13	GI	Cowden syndrome	303	Cowden syndrome/Multiple Hamartoma syndrome		
14	GI	Mycophenolate mofetil-associated colitis	684	Mycophenolate mofetil-associated colitis	Drug-induced colitis	
15	GI	Idiopathic mesenteric phlebosclerotic colitis	392	Idiopathic mesenteric phlebosclerotic colitis /Idiopathic Mesenteric Phlebosclerosis	Phlebosclerotic Colitis	
16	GI	Small bowel diaphragm disease	481	Small bowel diaphragm disease/NSAID-induced Small Intestinal Diaphragm Disease/ NSAID-induced diaphragm disease		Nonsteroidal anti-inflammatory drug (NSAID) enteropathy/ NSAID-induced enteropathy leading to intestinal strictures
17	GI	Gardner syndrome	530	Gardner syndrome		Familial adenomatous polyposis (FAP)
18	GI	Diffuse esophageal spasm	251	Diffuse esophageal spasm		
19	GI	Ménétrier's disease (Hypertrophic gastropathy or Hypertrophic gastritis)	631	Menetrier's disease	Hypertrophic gastropathy/ Hypertrophic gastritis	
20	GI	Abdominal cocoon syndrome	817	Abdominal cocoon/ sclerosing encapsulating peritonitis/ Encapsulating peritoneal sclerosis		
21	GI	Refractory diarrhea etiology: Clostridium	409	Clostridium difficile infection/	Pseudomembranous colitis	Medication-induced diarrhea/ Antibiotic-

		difficile infection			associated colitis,
22	GI	Lymphocytic esophagitis	343	Lymphocytic esophagitis		
23	GI	Hepatoid adenocarcinoma of the stomach	323	Hepatoid adenocarcinoma of the stomach /Gastric cancer (hepatoid adenocarcinoma subtype)		AFP-producing gastric carcinoma
24	GI	Gastric tuberculosis	563	Gastric tuberculosis	Reactivation of pulmonary tuberculosis	
25	GI	Gardner syndrome	443	Gardner syndrome		Familial adenomatous polyposis (FAP)
26	Genetics	Acute intermittent porphyria	477	Acute intermittent porphyria	Porphyria	
27	Genetics	Citrullinemia (Urea cycle disorder)	509	Urea cycle disorder/	Late-onset ornithine transcarbamylase (OTC) Deficiency	
28	Genetics	Abdominal pain etiology: Porphyria	378	Porphyria		
29	Genetics	Mitochondrial neurogastrointestinal encephalomyopathy	462	Mitochondrial neurogastrointestinal encephalomyopathy	Metabolic disorders/ Mitochondrial Myopathy/ Mitochondrial Disease	
30	Genetics	Hereditary angioedema	483	Hereditary angioedema		
31	Genetics	Left ventricular non-compaction cardiomyopathy	301	Left ventricular non-compaction cardiomyopathy	Myocardial Non-compaction Cardiomyopathy	
32	Genetics	Primary porphyria	715	Porphyria		
33	Genetics	Hereditary hemorrhagic telangiectasia	816	Hereditary hemorrhagic telangiectasia/ Osler-Weber-Rendu syndrome		

34	Hematology	Primary myelofibrosis	590	Primary Myelofibrosis	Myelofibrosis	Myeloproliferative disorders
35	Hematology	Multiple myeloma	410	Multiple myeloma/ Plasma Cell Myeloma		
36	Hematology	Primary non-Hodgkin lymphoma of the liver (Diffuse, T-cell phenotype)	803	Primary non-Hodgkin lymphoma of the liver	Lymphoma	
37	Hematology	Extranodal NK/T-cell lymphoma	910	Extranodal NK/T-cell lymphoma	Intestinal T-cell lymphoma/ Enteropathy-associated T-cell lymphoma/ lymphoma	
38	Hematology	Systemic mastocytosis (Involving the gastrointestinal tract)	411	Systemic mastocytosis/ Mastocytosis		
39	Hematology	Erythropoietic protoporphyria	651	Erythropoietic protoporphyria	Protoporphyria	
40	Hematology	Waldenström macroglobulinemia	395	Waldenström macroglobulinemia/ Lymphoplasmacytic Lymphoma		Lymphoma
41	Hematology	Multisystem Langerhans cell histiocytosis	882	Multisystem Langerhans cell histiocytosis /Langerhans cell histiocytosis		
42	Hematology	Small intestinal T-cell lymphoma	830	Small intestinal T-cell lymphoma	Lymphoma	
43	Immunology	Henoch-Schönlein purpura (Abdominal type)	655	Henoch-Schönlein Purpura		
44	Immunology	Antiphospholipid syndrome	580	Antiphospholipid syndrome		
45	Immunology	Felty's syndrome	794	Felty's syndrome/ Rheumatoid		Rheumatoid arthritis/

		(Rheumatoid arthritis-splenomegaly syndrome)		arthritis-splenomegaly syndrome		Rheumatoid arthritis with secondary splenomegaly
46	Immunology	Tacrolimus-induced vasculitis	614	Tacrolimus-induced vasculitis	Adverse reaction to tacrolimus and Vasculitis	Vasculitis/ Medication-induced Side Effects (Tacrolimus-related)
47	Immunology	Systemic lupus erythematosus	774	Systemic lupus erythematosus		
48	Immunology	Selective IgA deficiency (with Giardia infection)	588	Selective IgA deficiency (with Giardia infection)	Selective IgA deficiency	
49	Immunology	IgG4-related disease	755	IgG4-related disease		
50	Immunology	Congenital immunodeficiency disorders	452	Congenital immunodeficiency disorders	Common Variable Immunodeficiency	Immunodeficiency Disorders
51	Immunology	Eosinophilic granulomatosis with polyangiitis	554	Eosinophilic granulomatosis with polyangiitis/ Churg-Strauss Syndrome		Granulomatosis with polyangiitis/Microscopic polyangiitis
52	Immunology	Henoch-Schönlein purpura	578	Henoch-Schönlein purpura		
53	Multiple system	POEMS syndrome	583	POEMS syndrome		
54	Multiple system	Systemic sclerosis (CREST syndrome subtype)	921	Systemic sclerosis (CREST syndrome subtype)	Systemic sclerosis/ Scleroderma	
55	Multiple system	Paraneoplastic syndrome (Trousseau syndrome associated with metastatic	904	Paraneoplastic syndrome (Trousseau syndrome associated with metastatic cancer of unknown origin)/ Trousseau syndrome	Cancer with paraneoplastic syndrome leading to DIC and multiorgan dysfunction/ Metastatic cancer with	Non-Bacterial Thrombotic Endocarditis (NBTE) associated with an underlying metastatic

		cancer of unknown origin)			paraneoplastic syndrome	carcinoma/ Occult malignancy with an associated hypercoagulable state/ Metastatic Carcinoma with Associated Thromboembolism (Marantic Endocarditis)
56	Multiple system	Systemic primary amyloidosis	559	Systemic primary amyloidosis	Amyloidosis	
57	Multiple system	Primary amyloidosis (AL type)	858	Primary amyloidosis (AL type)	Amyloidosis	
58	Multiple system	Systemic sclerosis	691	Systemic sclerosis/ Scleroderma		
59	Multiple system	Light chain deposition disease of the small intestine	557	Light chain deposition disease/κ Light chain deposition disease		
60	Multiple system	Systemic light chain amyloidosis	511	Systemic light chain amyloidosis	Amyloidosis	
61	Multiple system	POEMS syndrome	613	POEMS syndrome		
62	Others	Renal tubular acidosis (Distal renal tubular acidosis)	691	Renal tubular acidosis (Distal renal tubular acidosis)/ Distal Renal Tubular Acidosis (Type I RTA)	Type IV renal tubular acidosis (RTA)	
63	Others	Hemophagocytic syndrome (Evolving from Crohn's	680	Hemophagocytic syndrome (Evolving from Crohn's disease)/ Hemophagocytic syndrome and	Hemophagocytic Lymphohistiocytosis (HLH)	

		disease)		Crohn's disease	
64	Others	Type I renal tubular acidosis (Distal renal tubular acidosis)	506	Type I renal tubular acidosis (Distal renal tubular acidosis)	
65	Others	Hemophagocytic syndrome (Evolving from colonic ulcers)	559	Hemophagocytic syndrome (Evolving from colonic ulcers)/	Hemophagocytic Lymphohistiocytosis (HLH)
66	Others	Lead poisoning (Causing recurrent pseudo-intestinal obstruction and anemia)	556	Lead poisoning	
67	Others	Hemorrhagic fever with renal syndrome	467	Hemorrhagic fever with renal syndrome	Hantavirus infection/ Hantavirus pulmonary syndrome

* Case reports were tokenized using byte pair encoding algorithm.

Supplementary Table 4. Diagnostic performance evaluation of physicians

Physician ID	Responses Number	Refusal Rate [*](%, 95%CI [**])	Average Number of Diagnoses (SD [**])	Coverage Rate (%, 95%CI)	Accuracy (%, 95%CI)	Average Time per Case (min)
**1**	67	0 (0-5.4)	1.9 (0.8)	34.3 (24.1-46.3)	25.4 (16.5-36.9)	3.7
**GI [**]**	25	0 (0 - 13.3)	1.7 (0.6)	28.0 (14.3 - 47.6)	24.0 (11.5 - 43.4)	-
**Non-GI**	42	0 (0 - 8.4)	1.9 (0.9)	38.1 (25.0 - 53.2)	26.2 (15.3 - 41.1)	-
**2**	67	6.0 (2.3-14.4)	1.3 (0.6)	26.9 (17.7-38.5)	26.9 (17.7-38.5)	6.9
**GI**	25	4.0 (0.7 - 19.5)	1.2 (0.4)	40.0 (23.4 - 59.3)	40.0 (23.4 - 59.3)	-
**Non-GI**	42	7.1 (2.5 - 19.0)	1.4 (0.7)	19.0 (10.0 - 33.3)	19.0 (10.0 - 33.3)	-
**3**	29	0 (0-11.7)	1.4 (0.6)	27.6 (14.7-45.7)	17.2 (7.6-34.6)	4.9
**GI**	11	0 (0 - 25.9)	1.5 (0.7)	45.5 (21.3 - 72.0)	36.4 (15.2 - 64.6)	-
**Non-GI**	18	0 (0 - 17.6)	1.4 (0.6)	16.7 (5.8 - 39.2)	5.6 (1.0 - 25.8)	-
**4**	67	0 (0-5.4)	1.6 (0.7)	50.8 (39.1-62.4)	40.3 (29.4-52.3)	5.2
**GI**	25	0 (0 - 13.3)	1.4 (0.6)	68.0 (48.4 - 82.8)	64.0 (44.5 - 79.8)	-
**Non-GI**	42	0 (0 - 8.4)	1.7 (0.7)	40.5 (27.0 - 55.5)	26.2 (15.3 - 41.1)	-
**5**	67	0 (0-5.4)	1.4 (0.7)	23.9 (15.3-35.3)	23.9 (15.3-35.3)	6
**GI**	25	0 (0 - 13.3)	1.3 (0.6)	32.0 (17.2 - 51.6)	32.0 (17.2 - 51.6)	-
**Non-GI**	42	0 (0 - 8.4)	1.5 (0.7)	19.0 (10.0 - 33.3)	19.0 (10.0 - 33.3)	-
**6**	67	0 (0-5.4)	1.3 (0.5)	47.8 (36.3-59.5)	38.8 (28.1-50.8)	4.9
**GI**	25	0 (0 - 13.3)	1.3 (0.5)	52.0 (33.5 - 70.0)	40.0 (23.4 - 59.3)	-
**Non-GI**	42	0 (0 - 8.4)	1.3 (0.5)	45.2 (31.2 - 60.1)	38.1 (25.0 - 53.2)	-
**7**	67	3.0 (0.2-10.9)	1.4 (0.7)	23.9 (15.3-35.3)	23.9 (15.3-35.3)	7.3
**GI**	25	0 (0 - 13.3)	1.4 (0.8)	20.0 (8.9 - 39.1)	20.0 (8.9 - 39.1)	-
**Non-GI**	42	0 (0 - 8.4)	1.4 (0.6)	26.2 (15.3 - 41.1)	26.2 (15.3 - 41.1)	-
**8**	67	3.0 (0.8-10.3)	1.5 (0.8)	38.8 (28.1-50.8)	32.8 (22.8-44.8)	13.9
**GI**	25	4.0 (0.7 - 19.5)	1.4 (0.6)	48.0 (30.0 - 66.5)	40.0 (23.4 - 59.3)	-
**Non-GI**	42	2.4 (0.4 - 12.3)	1.6 (0.9)	33.3 (21.0 - 48.4)	28.6 (17.2 - 43.6)	-
**9**	38	0 (0-9.2)	1.2 (0.4)	34.2 (21.2-50.1)	23.7 (13.0-39.2)	5.4
**GI**	16	0 (0 - 19.4)	1.1 (0.3)	56.2 (33.2 - 76.9)	43.8 (23.1 - 66.8)	-
**Non-GI**	22	0 (0 - 14.9)	1.2 (0.4)	18.2 (7.3 - 38.5)	9.1 (2.5 - 27.8)	-
**10**	67	0 (0-5.4)	1.4 (0.8)	23.9 (15.3-35.3)	20.9 (12.9-32.1)	9.8

**GI**	25	0 (0 - 13.3)	1.6 (0.9)	40.0 (23.4 - 59.3)	32.0 (17.2 - 51.6)	-
**Non-GI**	42	0 (0 - 8.4)	1.4 (0.7)	14.3 (6.7 - 27.8)	14.3 (6.7 - 27.8)	-
**11**	67	0 (0-5.4)	1.3 (0.7)	41.8 (30.7-53.7)	37.3 (26.7-49.3)	6.9
**GI**	25	0 (0 - 13.3)	1.2 (0.5)	56.0 (37.1 - 73.3)	48.0 (30.0 - 66.5)	-
**Non-GI**	42	0 (0 - 8.4)	1.4 (0.8)	22.2 (9.0 - 45.2)	31.0 (19.1 - 46.0)	-
**12**	29	0 (0-11.7)	1.4 (0.7)	24.1 (12.2-42.1)	17.2 (7.6-34.6)	3.2
**GI**	11	0 (0 - 25.9)	1.5 (0.9)	27.3 (9.7 - 56.6)	27.3 (9.7 - 56.6)	-
**Non-GI**	18	0 (0 - 17.6)	1.3 (0.6)	33.3 (21.0 - 48.4)	11.1 (3.1 - 32.8)	-
**13**	67	1.5 (0.3-8.0)	1.2 (0.7)	13.4 (7.2-23.6)	11.9 (6.2-21.8)	3.2
**GI**	25	0 (0 - 13.3)	1.3 (1.0)	8.0 (2.2 - 25.0)	8.0 (2.2 - 25.0)	-
**Non-GI**	42	2.4 (0.4 - 12.3)	1.1 (0.3)	16.7 (8.3 - 30.6)	14.3 (6.7 - 27.8)	-
**14**	67	0 (0-5.4)	1.1 (0.4)	17.9 (10.6-28.8)	16.4 (9.4-27.1)	2.8
**GI**	25	0 (0 - 13.3)	1.2 (0.4)	24.0 (11.5 - 43.4)	20.0 (8.9 - 39.1)	-
**Non-GI**	42	0 (0 - 8.4)	1.1 (0.4)	14.3 (6.7 - 27.8)	14.3 (6.7 - 27.8)	-
**15**	67	0 (0-5.4)	1.5 (0.7)	29.9 (20.2-41.7)	19.4 (11.7-30.4)	11.8
**GI**	25	0 (0 - 13.3)	1.6 (0.8)	32.0 (17.2 - 51.6)	24.0 (11.5 - 43.4)	-
**Non-GI**	42	0 (0 - 8.4)	1.5 (0.5)	28.6 (17.2 - 43.6)	16.7 (8.3 - 30.6)	-
**16**	67	1.5 (0.3-8.0)	1.5 (1.1)	41.8 (30.7-53.7)	32.8 (22.8-44.8)	10.5
**GI**	25	0 (0 - 13.3)	1.6 (1.2)	48.0 (30.0 - 66.5)	40.0 (23.4 - 59.3)	-
**Non-GI**	42	2.4 (0.4 - 12.3)	1.5 (1.0)	38.1 (25.0 - 53.2)	28.6 (17.2 - 43.6)	-
**17**	67	13.4 (7.2-23.6)	1.1 (0.4)	14.9 (8.3-25.3)	11.9 (6.2-21.8)	2.5
**GI**	25	8.0 (2.2 - 25.0)	1.1 (0.4)	24.0 (11.5 - 43.4)	20.0 (8.9 - 39.1)	-
**Non-GI**	42	16.7 (8.3 - 30.6)	1.1 (0.4)	9.5 (3.8 - 22.1)	7.1 (2.5 - 19.0)	-
**18**	67	12.0 (6.2-21.8)	1.5 (0.7)	25.4 (16.5-36.9)	23.9 (15.3-35.3)	2.9
**GI**	25	16.0 (6.4 - 34.7)	1.4 (0.6)	32.0 (17.2 - 51.6)	32.0 (17.2 - 51.6)	-
**Non-GI**	42	9.5 (3.8 - 22.1)	1.5 (0.8)	21.4 (11.7 - 35.9)	19.0 (10.0 - 33.3)	-
**19**	67	13.4 (7.2-23.6)	1.1 (0.4)	25.4 (16.5-36.9)	23.9 (15.3-35.3)	4.5
**GI**	25	12.0 (4.2 - 30)	1.1 (0.3)	20.0 (8.9 - 39.1)	20.0 (8.9 - 39.1)	-
**Non-GI**	42	14.3 (6.7 - 27.8)	1.1 (0.4)	28.6 (17.2 - 43.6)	26.2 (15.3 - 41.1)	-
**20**	48	0 (0-7.4)	2.5 (1.2)	39.6 (27.0-53.7)	27.1 (16.6-41.0)	20.4
**GI**	16	0 (0 - 19.4)	2.5 (1.2)	43.8 (23.1 - 66.8)	43.8 (23.1 - 66.8)	-

**Non-GI**	32	0 (0 - 10.7)	2.5 (1.1)	37.5 (22.9 - 54.7)	18.8 (8.9 - 35.3)	-
**21**	38	0 (0-9.2)	2.0 (0.7)	15.8 (7.4-30.4)	2.6 (0.5-13.5)	1.9
**GI**	13	0 (0 - 22.8)	2.2 (0.8)	23.1 (8.2 - 50.3)	7.7 (1.4 - 33.3)	-
**Non-GI**	25	0 (0 - 13.3)	1.9 (0.6)	12.0 (4.2 - 30.0)	0 (0 - 13.3)	-
**22**	29	0 (0-11.7)	1.4 (0.7)	13.8 (5.5-30.6)	13.8 (5.5-30.6)	2.5
**GI**	11	0 (0 - 25.9)	1.1 (0.3)	27.3 (9.7 - 56.6)	27.3 (9.7 - 56.6)	-
**Non-GI**	18	0 (0 - 17.6)	1.6 (0.8)	5.6 (1.0 - 25.8)	5.6 (1.0 - 25.8)	-
**Total**	1283	2.7 (1.9-3.6)	1.4 (0.7)	29.5 (27.1-32.1)	24.3 (22.0-26.7)	6.6
**GI**	478	2.3 (1.3-4.1)	1.4 (0.8)	36.2 (32.0-40.6)	31.6 (27.6-35.9)	-
**Non-GI**	805	2.9 (1.9-4.3)	1.5 (0.8)	25.6 (22.7-28.7)	20.0 (17.4-22.9)	-

* Refusing to respond the most likely diagnosis is regard as wrong in the estimation of coverage and accuracy.

** CI: confidence interval; SD: standard deviation; GI: Gastroenterology.

Supplementary Table 5. Correlation analysis between diagnostic coverage rate /accuracy and other variables

Diagnostic Measures	Variables	Correlation Coefficient	p value
**Coverage rate**	Token number of case record	-0.022	0.861
	Gender of physician (Female:1, Male:0)	0.166	0.461
	Age (31~40 years: 0, 41~50 years: 1, 51~60 years: 2,)	0.415	0.055
	Clinical experience (year)	0.385	0.076
	Monthly case load	-0.067	0.766
	Average spent time per question	**0.474**	**0.026**
	Percentage of using auxiliary methods	**0.522**	**0.013**
**Accuracy**	Token number of case record [a]	-0.084	0.500
	Gender (Female:1, Male:0)	0.181	0.419
	Age (31~40 years: 0, 41~50 years: 1, 51~60 years: 2,)	**0.542**	**0.009**
	Clinical experience (year)	**0.449**	**0.036**
	Monthly case load	-0.092	0.684
	Average spent time per question	0.399	0.066
	Percentage of using auxiliary methods	**0.538**	**0.010**

Supplementary Table 6. Correlation analysis between token numbers and judgment classification

LLMs	Token Numbers-Coverage judgment classification*		Token Numbers-Accuracy judgment classification*	
	Correlation Coefficient	p value	Correlation Coefficient	p value
**GPT 3.5t**	0.04	0.451	0.08	0.386
**GPT 4o**	0.10	0.312	0.14	0.244
**Gemini-1.0-pro**	0.12	0.337	0.09	0.367
**Gemini-1.5-pro**	0.04	0.614	0.03	0.745
**Claude 2.1**	-0.02	0.612	0.07	0.561
**Claude 3 Opus**	0.05	0.611	0.03	0.457
**Claude 3.5 Sonnet**	0.03	0.514	0.07	0.566
**Physician 1**	-0.01	0.941	0.08	0.513
**Physician 2**	0	0.977	0	0.977
**Physician 3**	0.26	0.174	0.24	0.219
**Physician 4**	0.15	0.222	0.19	0.124
**Physician 5**	0.07	0.554	0.07	0.554
**Physician 6**	0.03	0.801	0.05	0.667
**Physician 7**	0.01	0.927	0.01	0.927
**Physician 8**	0.03	0.814	0	0.997
**Physician 9**	0.49	**0.002**	0.42	**0.008**
**Physician 10**	0.13	0.312	0.09	0.449
**Physician 11**	0.19	0.114	0.18	0.149
**Physician 12**	0	0.993	0.19	0.318
**Physician 13**	-0.13	0.313	-0.1	0.415
**Physician 14**	0.05	0.684	0.02	0.861
**Physician 15**	-0.04	0.736	0.08	0.539
**Physician 16**	0.17	0.173	0.11	0.363
**Physician 17**	0.31	**0.011**	0.29	**0.017**
**Physician 18**	0.13	0.279	0.14	0.259
**Physician 19**	-0.05	0.707	-0.02	0.874

**Physician 20**	0.18	0.213	0.2	0.176
**Physician 21**	0.04	0.804	0.15	0.353
**Physician 22**	0.09	0.650	0.09	0.650

* Positive: 1, Negative:0, assessment criteria see Supplement Information 2

Supplementary Table 7. Diagnostic performance evaluation of LLMs in GI and non-GI Cases

Model	Disease Types	Accuracy (%, 95% CI*)	Coverage (%, 95% CI)
**GPT-3.5t**	GI*	3.0 (0.9 -8.6)	17.0 (10.6 -25.8)
	Non-GI*	7.7 (4.5 -12.9)	26.2 (20.0-33.5)
**GPT-4o**	GI	27.0 (18.9 -36.8)	59.0 (48.8 -68.5)
	Non-GI	52.4 (44.7-60.0)	67.3 (59.7-74.1)
**Gemini-1.0-pro**	GI	10.0 (5.3 -17.7)	24.0 (16.4 -33.6)
	Non-GI	20.2 (14.7-27.1)	34.5 (27.6-42.1)
**Gemini-1.5-pro**	GI	13.0 (7.5 -21.2)	48.0 (38.1 -58.1)
	Non-GI	31.5 (24.8-39.1)	56.5 (48.8-64.0)
**Claude-2.1**	GI	15.0 (9.0 -23.6)	38.0 (28.7 -48.1)
	Non-GI	26.8 (20.5-34.1)	41.7 (34.3-49.4)
**Claude 3 Opus**	GI	33.0 (24.2 -43.0)	51.0 (41.0 -60.9)
	Non-GI	51.2 (43.5-58.8)	75.6 (68.4-81.6)
**Claude 3.5 Sonnet**	GI	45.0 (35.2 -55.1)	73.0 (63.2 -81.1)
	Non-GI	51.2 (43.5-58.8)	78.0 (71.0-83.7)
**PULSE**	GI	0.0 (0.0 -3.8)	12.0 (6.8 -20.1)
	Non-GI	4.8 (2.4-9.2)	4.8 (2.4-9.2)

* CI: confidence interval; GI: Gastroenterology

Supplementary Table 8. Time and cost for various LLMs

LLMs	Spent Time Per Cases (sec)	Spent Fees Per Cases (USD)
**GPT 3.5t**	5.21 (1.30)	0.0020
**GPT 4o**	6.98 (2.26)	0.0074
**Gemini-1.0-pro**	4.26 (1.43)	0.0016
**Gemini-1.5-pro**	14.91 (3.67)	0.0053
**Claude 2.1**	14.94 (2.75)	0.0237
**Claude 3 Opus**	21.20 (3.04)	0.0552
**Claude 3.5 Sonnet**	10.09 (1.97)	0.0104

Supplementary Table 9. Occurrence of hallucinations in LLM responses across four test rounds

Model	Number of Responses with Hallucinations	Rate of Responses with Hallucinations (%)
**GPT-3.5t**	91	34.0
**Round 1**	21	31.3
**Round 2**	21	31.3
**Round 3**	23	34.3
**Round 4**	26	38.8
**GPT-4o**	62	23.1
**Round 1**	21	31.3
**Round 2**	13	19.4
**Round 3**	9	13.4
**Round 4**	19	28.4
**Gemini-1.0-pro**	89	33.2
**Round 1**	22	32.8
**Round 2**	21	31.3
**Round 3**	25	37.3
**Round 4**	21	31.3
**Gemini-1.5-pro**	168	62.7
**Round 1**	41	61.2
**Round 2**	46	68.7
**Round 3**	35	52.2
**Round 4**	36	53.7
**Claude-2.1**	149	55.6
**Round 1**	37	55.2
**Round 2**	42	62.7
**Round 3**	37	55.2
**Round 4**	33	49.3
**Claude 3 Opus**	79	29.5
**Round 1**	24	35.8
**Round 2**	23	34.3
**Round 3**	21	31.3
**Round 4**	11	16.4
**Claude 3.5 Sonnet**	57	21.3
**Round 1**	15	22.4
**Round 2**	10	14.9
**Round 3**	17	25.4
**Round 4**	15	22.4

Supplementary Table 10. Analysis of the correlation and odds ratio between hallucinations in LLM responses and erroneous responses

Model	GPT-3.5t	GPT-4o	Gemini-1.0-pro	Gemini-1.5-pro	Claude-2.1	Claude 3 Opus	Claude 3.5 Sonnet
**Comparison**	Hallucinations and errors in coverage rate evaluation						
**Phi coefficient**	0.128	0.032	0.000	0.080	0.008	0.017	0.040
**P-value of Phi coefficient**	0.039	0.606	1.000	0.193	0.891	0.783	0.509
**Odds Ratio***	0.516	0.815	1.019	1.439	1.068	1.125	1.325
**95% CI of Odds Ratio**	(0.287, 0.925)	(0.446, 1.489)	(0.586, 1.769)	(0.873, 2.373)	(0.654, 1.745)	(0.648, 1.953)	(0.684, 2.567)
**P-value of Odds Ratio**	0.026	0.505	0.948	0.154	0.793	0.677	0.404
**Comparison**	Hallucinations and errors in accuracy evaluation						
**Phi coefficient**	0.069	0.091	0.045	0.105	0.051	0.059	0.025
**P-value of Phi coefficient**	0.260	0.135	0.460	0.085	0.400	0.335	0.684
**Odds Ratio***	0.491	1.639	1.397	1.707	1.337	1.349	1.181
**95% CI of Odds Ratio**	(0.178, 1.355)	(0.905, 2.965)	(0.681, 2.865)	(0.971, 3.001)	(0.752, 2.378)	(0.791, 2.302)	(0.657, 2.124)
**P-value of Odds Ratio**	0.170	0.103	0.362	0.063	0.323	0.272	0.578

*Odds Ratio: Odds of errors in Coverage Rate/Accuracy evaluation given hallucinations

Supplementary Table 11. Error categorization of LLMs' responses

Model	Total Number of Errors	Error Types				
		Knowledge Deficiency	Ignoring Key Clues	Misinterpretation of Key Clues	Inadequate Diagnostic Reasoning	Refuse to Answer
GPT-3.5t	207	40	116	20	31	0
GPT-4o	96	4	70	18	4	0
Gemini-1.0-pro	186	62	101	15	8	0
Gemini-1.5-pro	125	20	65	22	18	0
Claude-2.1	160	12	122	23	3	0
Claude 3 Opus	90	0	50	35	5	0
Claude 3.5 Sonnet	64	0	25	35	4	0
PULSE	240	200	32	4	0	4

Percentage (%, 95% confidence interval)

	Total Number of Errors	Knowledge Deficiency	Ignoring Key Clues	Misinterpretation of Key Clues	Inadequate Diagnostic Reasoning	Refuse to Answer
GPT-3.5t	207	19.3 (14.4-25.3)	56.0 (49.1-62.8)	9.7 (6.3-14.5)	15.0 (10.7-20.6)	0.0 (0.0-1.8)
GPT-4o	96	4.2 (1.5-10.4)	72.9 (62.9-81.1)	18.8 (11.9-28.0)	4.2 (1.5-10.4)	0.0 (0.0-3.9)
Gemini-1.0-pro	186	33.3 (26.8-40.5)	54.3 (47.0-61.4)	8.1 (4.9-13.0)	4.3 (2.1-8.3)	0.0 (0.0-2.0)
Gemini-1.5-pro	125	16.0 (10.4-23.6)	52.0 (43.0-60.8)	17.6 (11.7-25.4)	14.4 (9.1-21.8)	0.0 (0.0-3.0)
Claude-2.1	160	7.5 (4.2-12.8)	76.3 (68.9-82.3)	14.4 (9.6-20.8)	1.9 (0.6-5.4)	0.0 (0.0-2.4)
Claude 3 Opus	90	0.0 (0.0-4.2)	55.6 (44.8-65.8)	38.9 (29.0-49.6)	5.6 (2.2-12.6)	0.0 (0.0-4.2)
Claude 3.5 Sonnet	64	0.0 (0.0-5.8)	39.1 (27.4-52.0)	54.7 (41.9-67.0)	6.3 (2.1-15.4)	0.0 (0.0-5.8)
PULSE	240	83.3 (78.0-87.6)	13.3 (9.5-18.3)	1.7 (0.6-4.2)	0.0 (0.0-1.6)	1.7 (0.6-4.2)

Supplementary Table 12. Baseline of the gastroenterologists attending questionnaire investigation

Number of Physician	Gender	Age (year)	Hospital Level	Subspecialty*	Clinical Experience (years)	Monthly Case Load	Number of Submitted Questionnaires	Error Analysis
**1**	Male	31~40	Tertiary	Pancreatobiliary diseases (including ERCP, EUS)	11	35	7	Yes
**2**	Male	31~40	Tertiary	Pancreatobiliary diseases (including ERCP, EUS), IBD, Small intestine disease	11	30	7	Yes
**3**	Male	41~50	Tertiary	ESD, STER, POEM	10	100	3	
**4**	Male	41~50	Tertiary	Pancreatobiliary diseases (including ERCP, EUS), Endoscopic Treatment of Varicose Veins, Gastrointestinal acute critical care	25	100	7	
**5**	Male	51~60	Tertiary	Hepatopancreatobiliary diseases (including ERCP, EUS), Gastrointestinal oncology	18	100	7	
**6**	Female	51~60	Tertiary	Pancreatobiliary diseases (including ERCP, EUS), Gastrointestinal motility disorder, ESD, STER, POEM, Endoscopic Treatment of Varicose Veins, Gastrointestinal oncology	25	0	7	
**7**	Female	41~50	Tertiary	Hepatic disease	17	90	7	Yes
**8**	Female	41~50	Tertiary	Pancreatobiliary diseases (including	20	40	7	Yes

				ERCP, EUS)				
**9**	Male	41~50	Tertiary	Pancreatobiliary diseases (including ERCP, EUS), IBD, Small intestine disease	23	50	4	
**10**	Male	41~50	Tertiary	General gastroenterology	19	70	7	
**11**	Male	51~60	Tertiary	ESD, STER, POEM, Pancreatobiliary diseases (including ERCP, EUS)	15	10	7	
**12**	Male	41~50	Tertiary	General gastroenterology	15	50	3	
**13**	Female	41~50	Tertiary	General gastroenterology	23	30	7	
**14**	Female	41~50	Tertiary	General gastroenterology, Pancreatobiliary diseases (including ERCP, EUS), Gastrointestinal acute critical care	23	60	7	
**15**	Female	31~40	Tertiary	General gastroenterology, Endoscopic Treatment of Varicose Veins	10	50	7	
**16**	Female	51~60	Tertiary	ESD, STER, POEM, Endoscopic Treatment of Varicose Veins	28	24	7	Yes
**17**	Female	31~40	Tertiary	Gastrointestinal acute critical care	10	70	7	
**18**	Female	41~50	Tertiary	Gastrointestinal oncology	19	13	7	
**19**	Male	41~50	Tertiary	Hepatic disease, Complex digestive diseases	16	200	7	

**20**	Male	41~50	Tertiary	ESD, STER, POEM, Hepatopancreatobiliary diseases (including ERCP, EUS), Endoscopic Treatment of Varicose Veins, Gastrointestinal oncology, Gastrointestinal acute critical care	22	140	5
**21**	Female	31~40	Tertiary	General gastroenterology, IBD, Small intestine disease, Gastrointestinal acute critical care	11	60	4
**22**	Male	41~50	Tertiary	Pancreatobiliary diseases (including ERCP, EUS), Endoscopic Treatment of Varicose Veins, Gastrointestinal oncology	20	25	3
**23**	Male	41~50	Tertiary	ESD, STER, POEM, Pancreatobiliary diseases (including ERCP, EUS)	20	80	2
**24**	Female	31~40	Tertiary	General gastroenterology	10	50	2
**25**	Female	51~60	Tertiary	General gastroenterology	39	50	1
**26**	Female	51~60	Tertiary	Pancreatobiliary diseases (including ERCP, EUS)， Endoscopic Treatment of Varicose Veins, IBD and Small intestine disease, Gastrointestinal rare disease, Gastrointestinal acute critical care	30	330	2
**27**	Male	41~50	Tertiary	Pancreatobiliary diseases (including ERCP, EUS), IBD, and Small intestine disease	24	60	2

**28**	Male	41~50	Tertiary	Pancreatobiliary diseases (including ERCP, EUS)	25	80	1
**29**	Female	41~50	Tertiary	ESD, STER, POEM, Pancreatobiliary diseases (including ERCP, EUS), Gastrointestinal rare disease, IBD	22	200	1
**30**	Female	41~50	Tertiary	Pancreatobiliary diseases (including ERCP, EUS)	15	30	2

* ESD: Endoscopic Submucosal Dissection, ERCP: Endoscopic Retrograde Cholangiopancreatography, EUS: Endoscopic Ultrasound, STER: Submucosal Tunneling Endoscopic Resection, POEM: Peroral Endoscopic Myotomy, IBD: Inflammatory Bowel Disease.

Supplementary Table 13. Error categorization of Physicians' responses

Error Types	Total	Knowledge Deficiency	Ignoring Key Clues	Misinterpretation of Key Clues	Inadequate Diagnostic Reasoning	Refuse to Answer
**Total Number of Errors**	224	144	24	35	18	3
**Errors in GI Cases**	79	39	12	17	9	2
**Errors in Non-GI Cases**	145	105	12	18	9	1

Error Types	Total	Percentage (%, 95% confidence interval)				
		**Knowledge Deficiency**	**Ignoring Key Clues**	**Misinterpretation of Key Clues**	**Inadequate Diagnostic Reasoning**	**Refuse to Answer**
**Total Number of Errors**	224	64.3 (57.7-70.4)	10.7 (7.2-15.5)	15.6 (11.4-21.0)	8.0 (5.1-12.4)	1.3 (0.4-3.9)
**Errors in GI Cases**	79	49.4 (38.1-60.7)	15.2 (8.5-25.1)	21.5 (13.5-32.2)	11.4 (5.8-20.6)	2.5 (0.5-9.0)
**Errors in Non-GI Cases**	145	72.4 (64.4-79.2)	8.3 (4.7-14.0)	12.4 (7.9-18.9)	6.2 (3.2-11.5)	0.7 (0.1-3.9)

Supplementary Table 14. Diagnostic performance of LLMs when only providing admission information and routine laboratory test results

	Coverage Rate (95%CI)	Accuracy (95%CI)
**GPT-4o**	38.8(33.1-44.8)	19.8(15.4-25.0)
**Round 1**	37.3(26.1-49.9)	22.4(13.5-34.3)
**Round 2**	34.3(23.5-46.9)	14.9(7.8-25.8)
**Round 3**	43.3(31.4-55.9)	19.4(11.2-31.0)
**Round 4**	40.3(28.7-52.9)	22.4(13.5-34.3)
**Gemini-1.5-pro**	33.6(28.1-39.5)	13.1(9.5-17.7)
**Round 1**	32.8(22.2-45.4)	14.9(7.8-25.8)
**Round 2**	38.8(27.4-51.4)	11.9(5.7-22.3)
**Round 3**	28.4(18.4-40.7)	11.9(5.7-22.3)
**Round 4**	34.3(23.5-46.9)	13.4(6.8-24.1)
**Claude 3.5 Sonnet**	45.1(39.2-51.2)	22.8(18.1-28.2)
**Round 1**	44.8(32.8-57.3)	20.9(12.3-32.6)
**Round 2**	43.3(31.4-55.9)	22.4(13.5-34.3)
**Round 3**	46.3(34.2-58.7)	26.9(17.1-39.1)
**Round 4**	46.3(34.2-58.7)	20.9(12.3-32.6)

Supplementary Table 15. Performance of five LLMs on the NEJM dataset

Models	Coverage Rate (%, 95% CI)	Accuracy (%, 95% CI)
GPT-3.5t	31.2 (27.8-34.8)	6.8 (5.1-8.9)
Round 1	30.6 (24.0-38.0)	7.6 (4.4-12.7)
Round 2	29.4 (22.9-36.8)	9.4 (5.8-14.8)
Round 3	32.4 (25.6-39.9)	4.7 (2.3-9.1)
Round 4	32.4 (25.6-39.9)	5.3 (2.7-9.8)
GPT-4o	48.2 (44.5-52.0)	21.8 (18.8-25.0)
Round 1	48.2 (40.7-55.9)	22.9 (17.1-30.0)
Round 2	50.0 (42.4-57.6)	22.4 (16.6-29.3)
Round 3	48.2 (40.7-55.9)	21.8 (16.1-28.7)
Round 4	46.4 (39.0-54.1)	20.0 (14.5-26.8)
Gemini-1.5-pro	50.4 (46.7-54.2)	19.4 (16.6-22.6)
Round 1	51.8 (44.1-59.3)	19.4 (14.0-26.1)
Round 2	49.4 (41.8-57.0)	20.0 (14.5-26.8)
Round 3	51.2 (43.6-58.7)	18.8 (13.5-25.5)
Round 4	49.4 (41.8-57.0)	19.4 (14.0-26.1)
Claude 3 Opus	58.4 (54.6-62.1)	27.6 (24.4-31.1)
Round 1	62.4 (54.7-69.5)	31.8 (25.0-39.3)
Round 2	65.9 (58.3-72.7)	31.2 (24.5-38.6)
Round 3	52.4 (44.7-59.9)	23.5 (17.6-30.6)
Round 4	52.9 (45.3-60.5)	24.1 (18.2-31.2)
Claude 3.5 Sonnet	68.2 (64.6-71.6)	32.8 (29.4-36.4)
Round 1	68.8 (61.4-75.5)	31.8 (25.1-39.3)
Round 2	67.6 (60.1-74.4)	32.9 (26.2-40.5)
Round 3	68.2 (60.7-74.9)	33.5 (26.7-41.1)
Round 4	68.2 (60.7-74.9)	32.9 (26.2-40.5)

Supplementary Table 16. Performance of five LLMs on the NEJM dataset patients with primary GI and Non-GI symptoms

Models	Primary Symptoms	Coverage Rate (%, 95%CI)	Accuracy (%, 95%CI)
**GPT-3.5t**	GI	25.8 (18.7-34.4)	3.2 (1.6-9.2)
	Non-GI	32.4 (28.6-36.4)	7.6 (2.6-10.1)
**GPT-4o**	GI	44.4 (35.6-53.4)	16.1 (10.5-23.8)
	Non-GI	49.1 (44.9-53.3)	23.0 (19.7-26.7)
**Gemini-1.5-pro**	GI	48.4 (39.5-57.4)	16.1 (10.5-23.8)
	Non-GI	50.9 (46.7-55.1)	20.1 (17.0-23.7)
**Claude 3 Opus**	GI	54.8 (45.8-63.6)	24.2 (17.3-32.7)
	Non-GI	59.2 (55.0-63.2)	28.4 (24.8-32.3)
**Claude 3.5 Sonnet**	GI	57.3 (48.2-65.9)	27.4 (20.1-36.1)
	Non-GI	70.7 (66.7-74.3)	34.0 (30.2-38.1)

Supplementary Table 17. Baseline of the members in Panel A and Panel B

Number of Physician	Gender	Age (year)	Hospital Level	Subspecialty	Clinical Experience (years)
**Panel A-1**	Male	41~50	Tertiary	ESD*; Gastrointestinal oncology	17
**Panel A-2**	Female	41~50	Tertiary	Pancreatobiliary diseases (including ERCP*, EUS*); Gastrointestinal acute critical care	21
**Panel A-3**	Female	41~50	Tertiary	Hepatopancreatobiliary diseases (including ERCP, EUS);	18
**Panel A-4**	Male	31~40	Tertiary	Hepatic disease	13
**Panel A-5**	Male	31~40	Tertiary	Gastrointestinal surgery	8
**Panel A-6**	Female	41~50	Tertiary	Gastrointestinal oncology; Small intestine disease	16
**Panel A-7**	Female	31~40	Tertiary	Gastrointestinal pathology	7
**Panel B-1**	Male	41~50	Tertiary	Pancreatobiliary diseases (including ERCP, EUS), Gastrointestinal motility disorder	21
**Panel B-2**	Male	51~60	Tertiary	Gastrointestinal oncology	32
**Panel B-3**	Male	41~50	Tertiary	Pancreatobiliary diseases (including ERCP); Enteroscopy; Small intestine disease	25

* ESD: Endoscopic Submucosal Dissection, ERCP: Endoscopic Retrograde Cholangiopancreatography, EUS: Endoscopic Ultrasound.

Supplementary Table 18. Model information and query date of the involved LLMs

LLMs *	Specific Model Number	Query Date	API Channel
**GPT 3.5t**	gpt-3.5-turbo-0613	October 1-5, 2024	Azure
**GPT 4o**	gpt-4o-2024-08-06	October 1-5, 2024	Azure
**Gemini-1.0-pro**	gemini-1.0-pro	October 1-5, 2024	Google Cloud Platform
**Gemini-1.5-pro**	gemini-1.5-pro-latest	October 1-5, 2024	Google Cloud Platform
**Claude 2.1**	Unknown	October 1-5, 2024	Anthropic
**Claude 3 Opus**	claude-3-opus-20240229	October 1-5, 2024	Google Cloud Platform
**Claude 3.5 Sonnet**	claude-3-5-sonnet-20240620	October 1-5, 2024	Google Cloud Platform

* All LLMs were accessed in Japan.

Supplementary Table 19. The coverage rate, accuracy and consistency evaluation of PULSE

Model	Average Number of Diagnoses (SD)	Accuracy (%, 95% CI)	Coverage Rate (%, 95% CI)	Krippendorff's Alpha
PULSE	4.1(1.7)	3.0 (1.5-5.8)	7.5 (4.8-11.3)	1.000
Round 1	4.1(1.7)	3.0 (0.6-10.5)	7.5 (2.9-16.7)	
Round 2	4.1(1.8)	3.0 (0.6-10.5)	7.5 (2.9-16.7)	
Round 3	4.1(1.6)	3.0 (0.6-10.5)	7.5 (2.9-16.7)	
Round 4	4.1(1.7)	3.0 (0.6-10.5)	7.5 (2.9-16.7)	

Supplementary Figure 1. Diagnostic performance, statistical significance and consistency analysis of 7 different LLMs across four query rounds

(a)

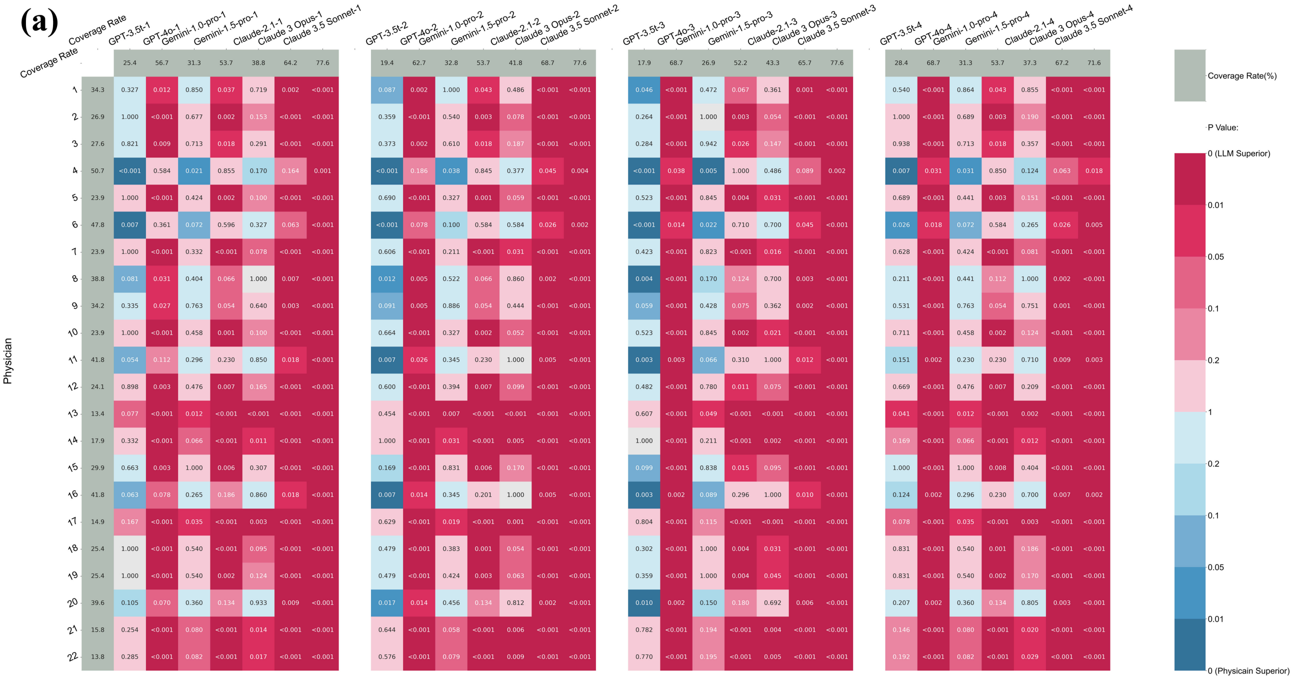

(b)

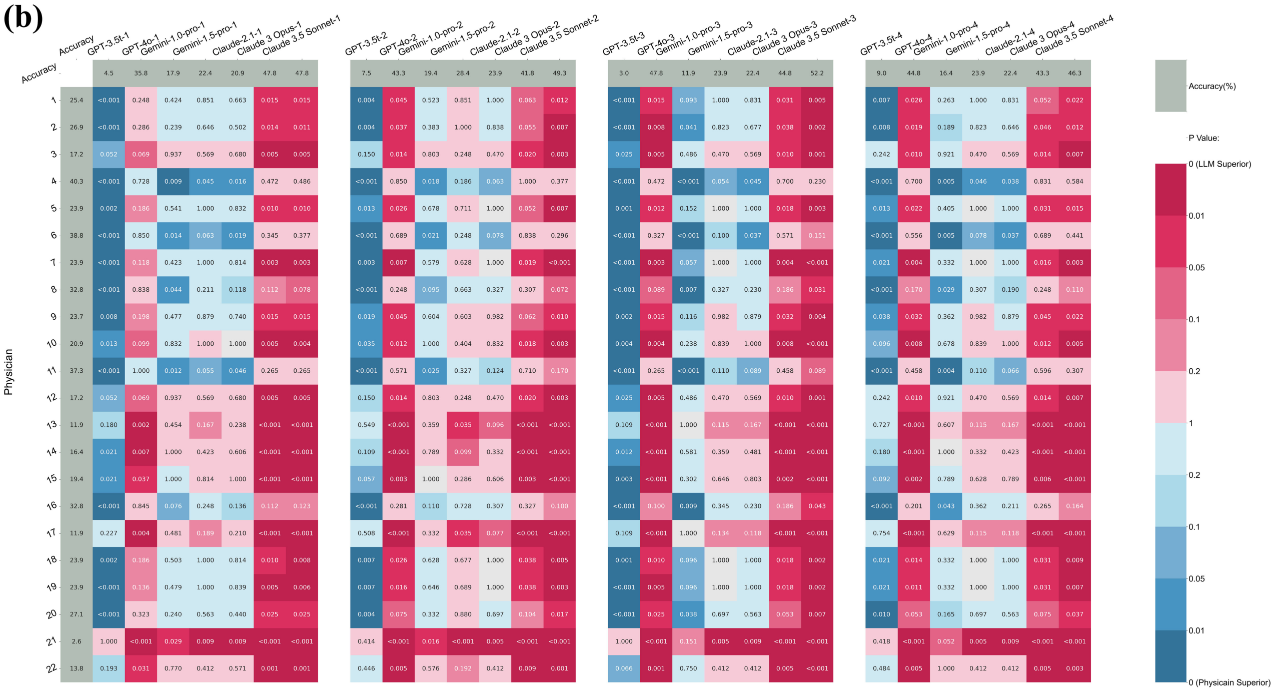

a. The heatmap consists of coverage rate of each LLM's responses in each query round and corresponding p values of McNemar’s significance tests.

b. The heatmap illustrates the accuracy of each LLM's responses in each query round and corresponding p values of McNemar’s significance tests.

Supplementary Figure 2. Statistical significance of the coverage rate and the accuracy between the LLMs in each round and the physicians

(a)

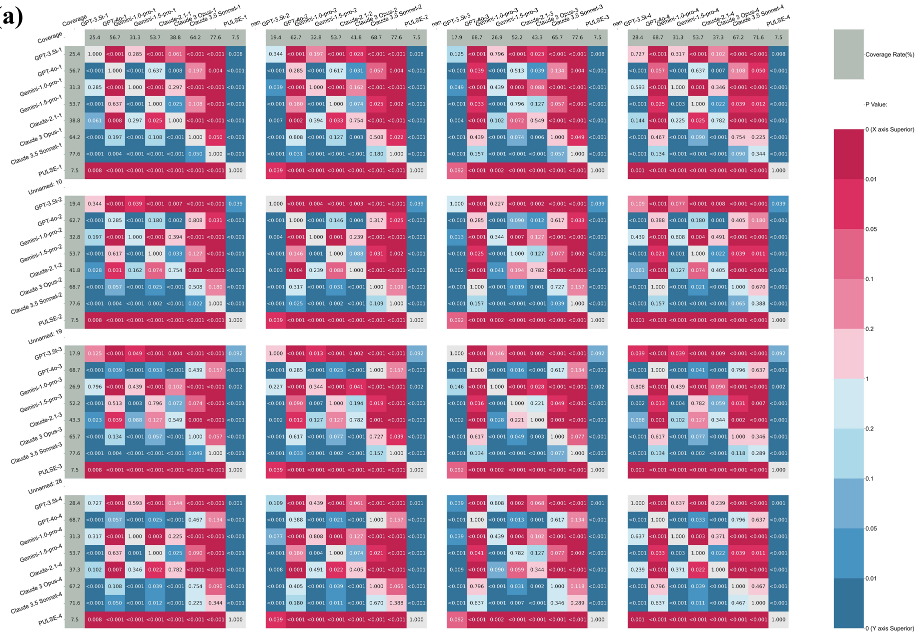

(b)

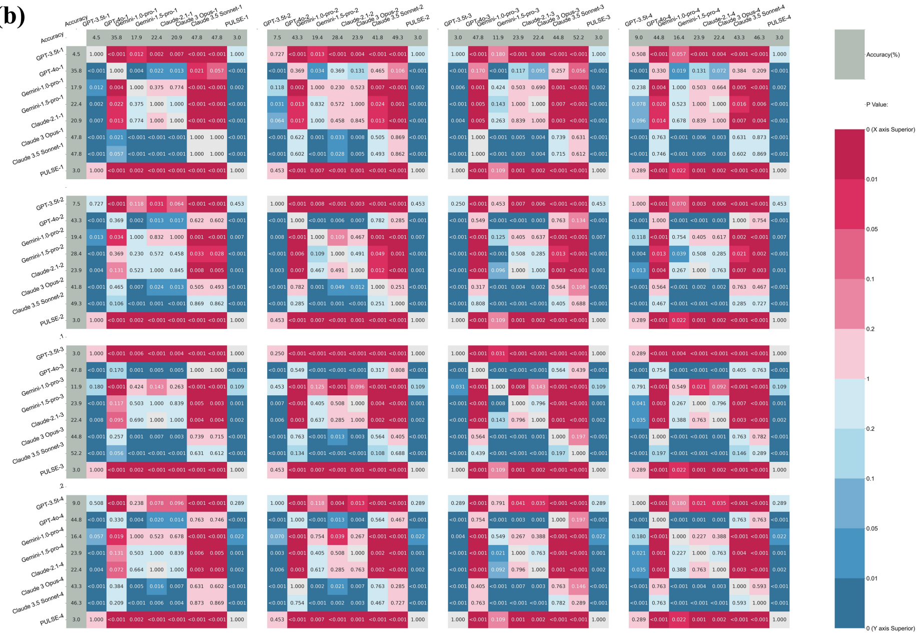

a. The heatmap shows the statistical significance of the coverage rate between the LLMs and the physicians.

b. The heatmap shows the statistical significance of the accuracy between the LLMs and the physicians.

Supplementary Figure 3. Statistical significance of the coverage rate and the accuracy between different LLMs in the GI and non-GI case subgroups

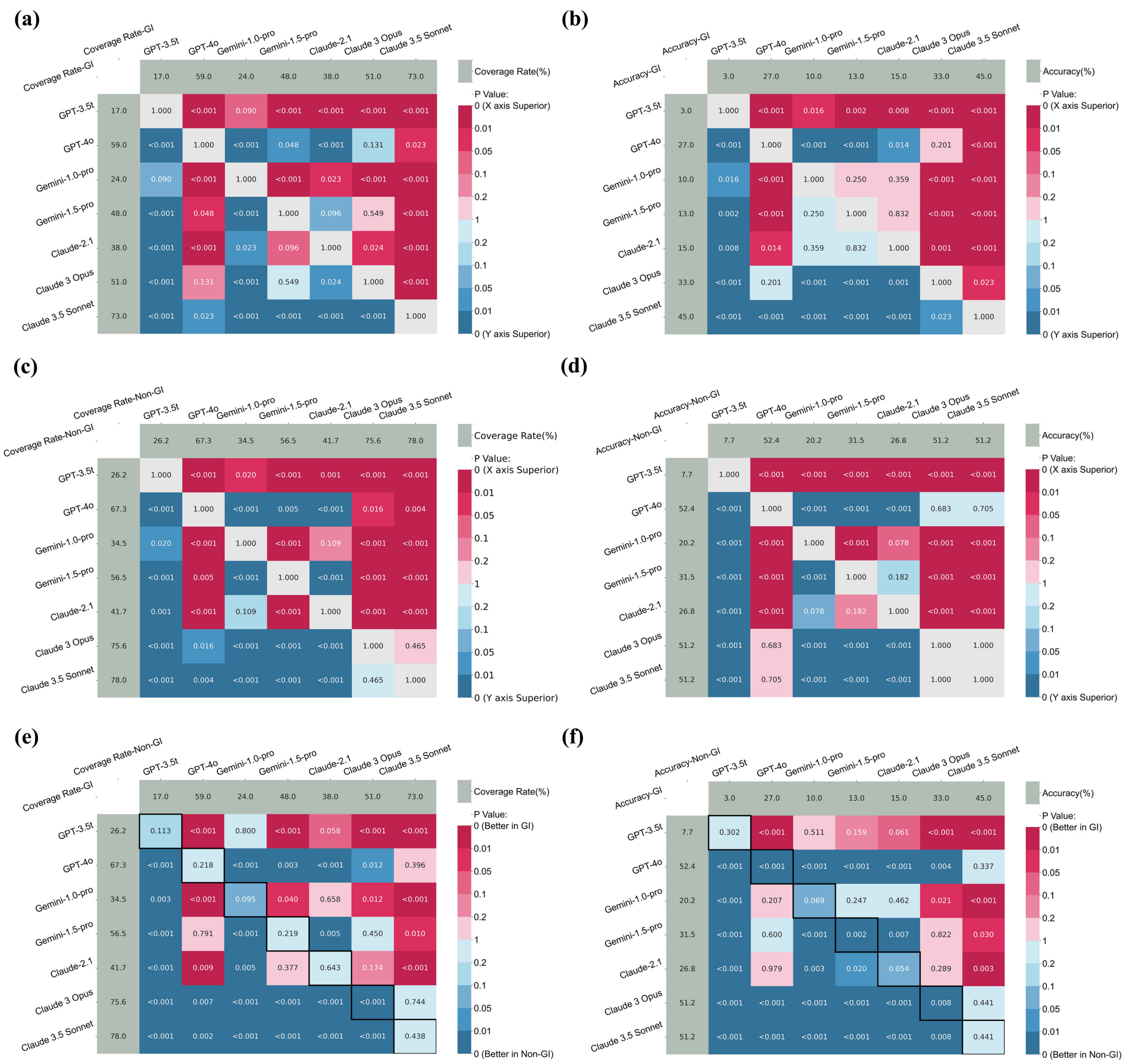

a, b. The two heatmaps show the statistical significance of the coverage rate and the accuracy between different LLMs in the GI case subgroup, respectively.

c, d. The heatmaps shows the statistical significance of the coverage rate and the accuracy between different LLMs in the non-GI case subgroup, respectively.

e, f. The heatmap shows the statistical significance of the coverage rate and the accuracy of each LLM between the GI and non-GI case subgroups, respectively.

Supplementary Figure 4. The comparison of PULSE with other closed-source LLMs or the performance of the physicians

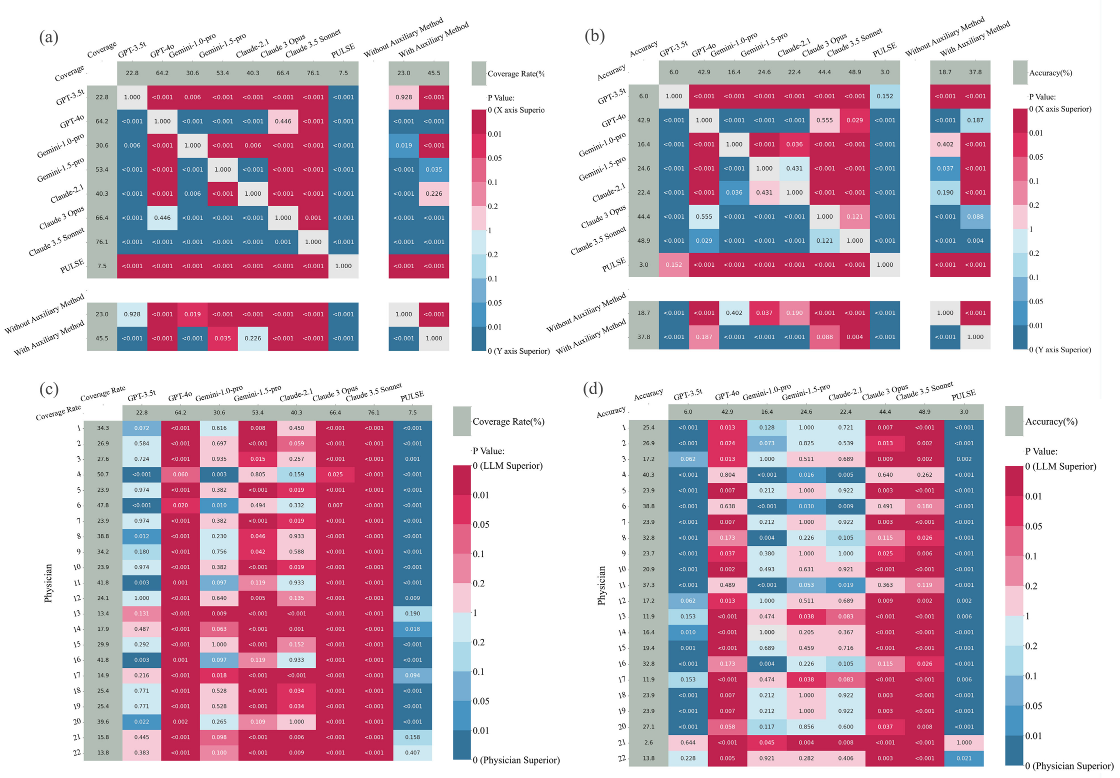

a-d The four heatmaps show the comparative diagnostic performance after incorporating PULSE. According to the differences in coverage rates and accuracies displayed in the graphs, PULSE demonstrates lower diagnostic performance in the offline dataset compared to other LLMs and the 18 gastroenterologists.

Reference

1. Zhang, X., Xue, K. & Zhang, S. PULSE: Pretrained and Unified Language Service Engine. (2023)
